# Detoxified synthetic bacterial membrane vesicles as a vaccine platform against bacteria and SARS-CoV-2

**DOI:** 10.1186/s12951-023-01928-w

**Published:** 2023-05-19

**Authors:** Kyong-Su Park, Kristina Svennerholm, Rossella Crescitelli, Cecilia Lässer, Inta Gribonika, Mickael Andersson, Jonas Boström, Hanna Alalam, Ali M Harandi, Anne Farewell, Jan Lötvall

**Affiliations:** 1 Krefting Research Centre, Institute of Medicine, Sahlgrenska Academy, University of Gothenburg, Gothenburg, Sweden; 2 Department of Anesthesiology and Intensive Care Medicine, Institute of Clinical Science, Sahlgrenska Academy, University of Gothenburg, Gothenburg, Sweden; 3 Department of Surgery, Institute of Clinical Sciences, Sahlgrenska Academy, University of Gothenburg, Gothenburg, Sweden; 4 Department of Surgery, Sahlgrenska University Hospital, Region Västra Götaland, Gothenburg, Sweden; 5 Wallenberg Centre for Molecular and Translational Medicine, University of Gothenburg, Gothenburg, Sweden; 6 Department of Microbiology and Immunology, Institute of Biomedicine, University of Gothenburg, Gothenburg, Sweden; 7 Department of Chemistry and Molecular Biology, Centre for Antibiotic Resistance, University of Gothenburg, Gothenburg, Sweden; 8 BC Children’s Hospital Research Institute, Vaccine Evaluation Center, University of British Columbia, Columbia, Canada

**Keywords:** Synthetic bacterial vesicles, Outer membrane vesicles, Vaccine, Pulmonary inflammation, Sepsis, COVID-19

## Abstract

The development of vaccines based on outer membrane vesicles (OMV) that naturally bud off from bacteria is an evolving field in infectious diseases. However, the inherent inflammatory nature of OMV limits their use as human vaccines. This study employed an engineered vesicle technology to develop synthetic bacterial vesicles (SyBV) that activate the immune system without the severe immunotoxicity of OMV. SyBV were generated from bacterial membranes through treatment with detergent and ionic stress. SyBV induced less inflammatory responses in macrophages and in mice compared to natural OMV. Immunization with SyBV or OMV induced comparable antigen-specific adaptive immunity. Specifically, immunization with *Pseudomonas aeruginosa*-derived SyBV protected mice against bacterial challenge, and this was accompanied by significant reduction in lung cell infiltration and inflammatory cytokines. Further, immunization with *Escherichia coli*-derived SyBV protected mice against *E. coli* sepsis, comparable to OMV-immunized group. The protective activity of SyBV was driven by the stimulation of B-cell and T-cell immunity. Also, SyBV were engineered to display the SARS-CoV-2 S1 protein on their surface, and these vesicles induced specific S1 protein antibody and T-cell responses. Collectively, these results demonstrate that SyBV may be a safe and efficient vaccine platform for the prevention of bacterial and viral infections.

**Supplementary Information:**

The online version contains supplementary material available at 10.1186/s12951-023-01928-w.

## Introduction

The prevention of bacterial infections of public health concern remains an unmet clinical need, and only a few preventive vaccines exist. It is especially difficult to treat *Pseudomonas aeruginosa*-related lung diseases due to the occurrence of multiple-drug resistant strains [1]. Indeed, multiple types of bacteria have developed multi-drug resistance and are contributing to a silent pandemic [2]. Thus, the development of a more effective vaccine platform is needed, and nanosized outer membrane vesicles (OMV) from Gram-negative bacteria have shown promise in this regard [3, 4].

Naturally produced OMV are spherical membrane bilayer proteolipid structures with a diameter of approximately 20–200 nm, and they carry essential molecules such as lipopolysaccharide (LPS) and other outer membrane proteins that are recognized by Toll-like receptors (TLRs) on immune cells [5, 6]. Due to their immunostimulatory potential, OMV have been shown to be very effective as adjuvants for the treatment of bacterial infections. Indeed, it has been confirmed that active immunization with purified *P. aeruginosa* OMV is able to reduce bacterial colonization, pro-inflammatory cytokine release, and lung tissue damage from *P. aeruginosa* infection in mice [7].

A major concern is that OMV are highly toxic due to their high concentration of LPS and other TLR-activating molecules, and OMV can induce severe inflammation in vivo such as sepsis, cardiac dysfunction, and acute lung disease through excessive activation of innate immune cells, leading sometimes to death [8–10]. We have recently developed detoxified OMV-like vesicles from *Escherichia coli*, named synthetic bacterial vesicles (SyBV), and have shown that these engineered vesicles, in contrast to natural OMV, induce limited or no systemic inflammation [11].

In the current study, we hypothesized that SyBV can be directly produced from *P. aeruginosa* membranes through the established process, including treatment with detergent and induction of ionic stress, and that these SyBV can be employed as a new vaccine platform to specifically induce protective immunity against *P. aeruginosa*-induced pneumonia. To test this hypothesis, we produced SyBV from *E. coli* and their protective efficacy and immunogenicity were determined in an experimental mouse sepsis model of *E. coli* infection. We also tested whether SyBV can be engineered to carry the SARS-CoV-2 S1 protein and whether immunization with S1-loaded SyBV can provide immune responses against the viral protein.

## Results

### ***P. aeruginosa*** SyBV were generated with higher yield and purity than natural OMV

**Fig. 1:**
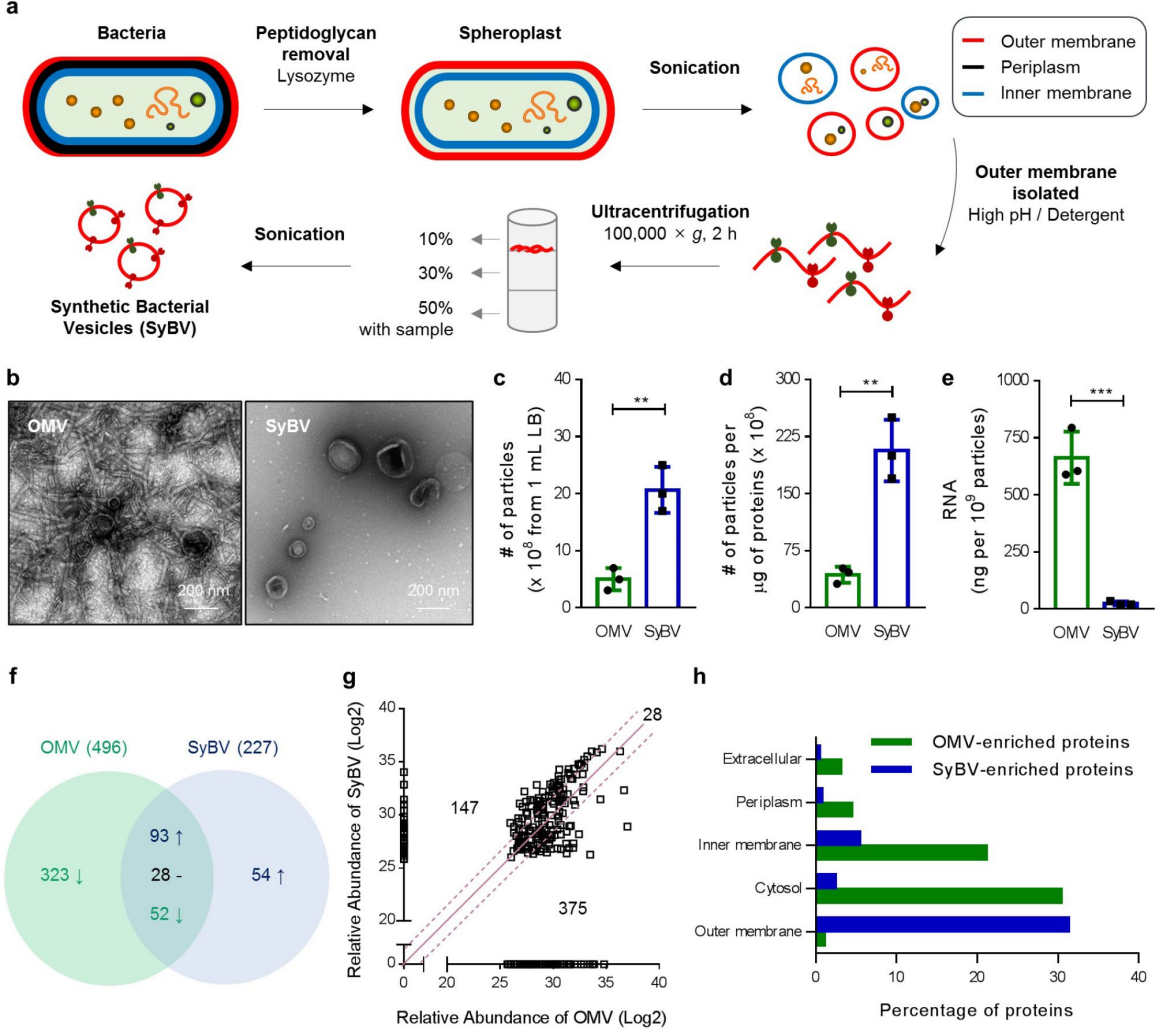
Isolation and characterization of *P. aeruginosa*-derived SyBV and OMV. **a** Schematic diagram of the generation of *P. aeruginosa* SyBV. **b** Representative TEM images of *P. aeruginosa* OMV and SyBV. Scale bars, 200 nm. **c-e** The number of particles derived from 1 mL Luria-Bertani culture medium (**c**), the number of particles per one microgram of proteins (**d**), and the total RNA amount included in one OMV and SyBV particle (**e**; *n* = 3 independent samples). Data are presented as the mean ± s.e.m. ^**^P < 0.01, ^***^P < 0.001 by unpaired two-tailed Student’s *t*-test. **f** Venn diagram of *P. aeruginosa* OMV and SyBV proteomes. The mutual proteins are divided into three groups (1.5-fold increase, 1.5-fold decrease, and no change) based on the relative protein abundance (*n* = 2 independent samples). The up arrow means identified proteins in only SyBV group or 1.5-fold increased proteins in SyBV group compared to OMV. In contrast, the down arrow means identified protein in only OMV group or 1.5-fold decreased proteins in SyBV group compared OMV. **g** Plot of the log2 value of the relative protein abundance from OMV and SyBV. The solid purple line and dotted lines indicate no change and 1.5-fold change, respectively. **h** OMV-enriched proteins and SyBV-enriched proteins were analyzed with by GO cellular component annotations

*P. aeruginosa* SyBV were directly produced from bacterial pellets according to the protocol indicated in Fig. 1a. Briefly, bacteria cells were treated with lysozyme to extract the periplasm and then sonicated to disrupt the cell wall. The resulting membranes were subjected to treatment with Sarkosyl detergent to remove bacterial inner membranes. The remaining membranes were then exposed to high pH to generate outer membrane sheets. The clean membrane structures were acquired from the interface layer of 10% and 30% iodixanol after buoyant density-gradient ultracentrifugation, after which SyBV were generated by sonication of the collected outer membranes. Natural OMV were also isolated from the *P. aeruginosa* culture supernatant as described in our previous study [10] and used for comparison with SyBV (Additional file 1: Fig. S1). Transmission electron microscopy (TEM) analysis of SyBV showed that they were closed spherical vesicles 50–150 nm in diameter, similar to natural OMV (Fig. 1b). However, unlike OMV, the SyBV did not show the pili-like structures in TEM images. Also, the SyBV were produced at a concentration 3 times higher than OMV from the same volume of culture medium (Fig .1c) and were 5 times purer in terms of the particle number per µg of protein, which was in line with the TEM results showing uncontaminated vesicles (Fig. 1d).

We next investigated the differences in composition of SyBV vs. natural OMV. Importantly, the OMV contained large quantities of RNA (Fig. 1e) and different sized peaks (Additional file 1: Fig. S2), whereas SyBV did not, indicating that the RNA was almost completely removed during the creation of the SyBV. No DNA was also detected in the SyBV from *P. aeruginosa* (Additional file 1: Fig. S3). This is important because the presence of bacterial RNA and DNA can activate nucleic acid-recognizing pattern-recognition receptors such as TLR3 or TLR9 in immune cells, leading to excessive inflammation [12]. We next compared the protein composition of SyBV and OMV by quantitative proteomics analysis. Principal component analysis showed that the first component separated 73% of the data based on vesicle type (SyBV and OMV), while the second component separated 9% of the data by replicate (Additional file 1: Fig. S4a). Hierarchical cluster analysis produced similar results, with vesicle samples grouping first by the type of vesicles and then by biological replicate (Additional file 1: Fig. S4b), suggesting that there are distinct protein profiles for each vesicle sample and that the repeated experiments yield comparable results. In total, 496 and 227 proteins were identified by mass spectrometry from OMV and SyBV, respectively (Fig. 1f). A total of 173 proteins were identified in both vesicle samples, whereas 323 and 54 proteins were exclusively identified in OMV and SyBV, respectively. Based on the relative protein abundance, the quantity of 28 proteins did not clearly change; however, 147 and 375 proteins were relatively increased and decreased in SyBV compared to OMV, respectively (Fig. 1g). The subcellular localization of the enriched proteins in OMV and SyBV was examined by the Gene Ontology (GO) term analysis (Fig. 1h), and the relatively high number of cytosolic proteins in OMV were absent in the SyBV proteome. Moreover, SyBV were enriched in outer membrane proteins that are related to strong immunogenicity [13]. Collectively, these findings indicate that the platform for the development of SyBV yields high amounts of pure vesicles with very few cytosolic contaminants.

### ***P. aeruginosa*** SyBV induce less toxicity than OMV with regards to inflammation

**Fig. 2:**
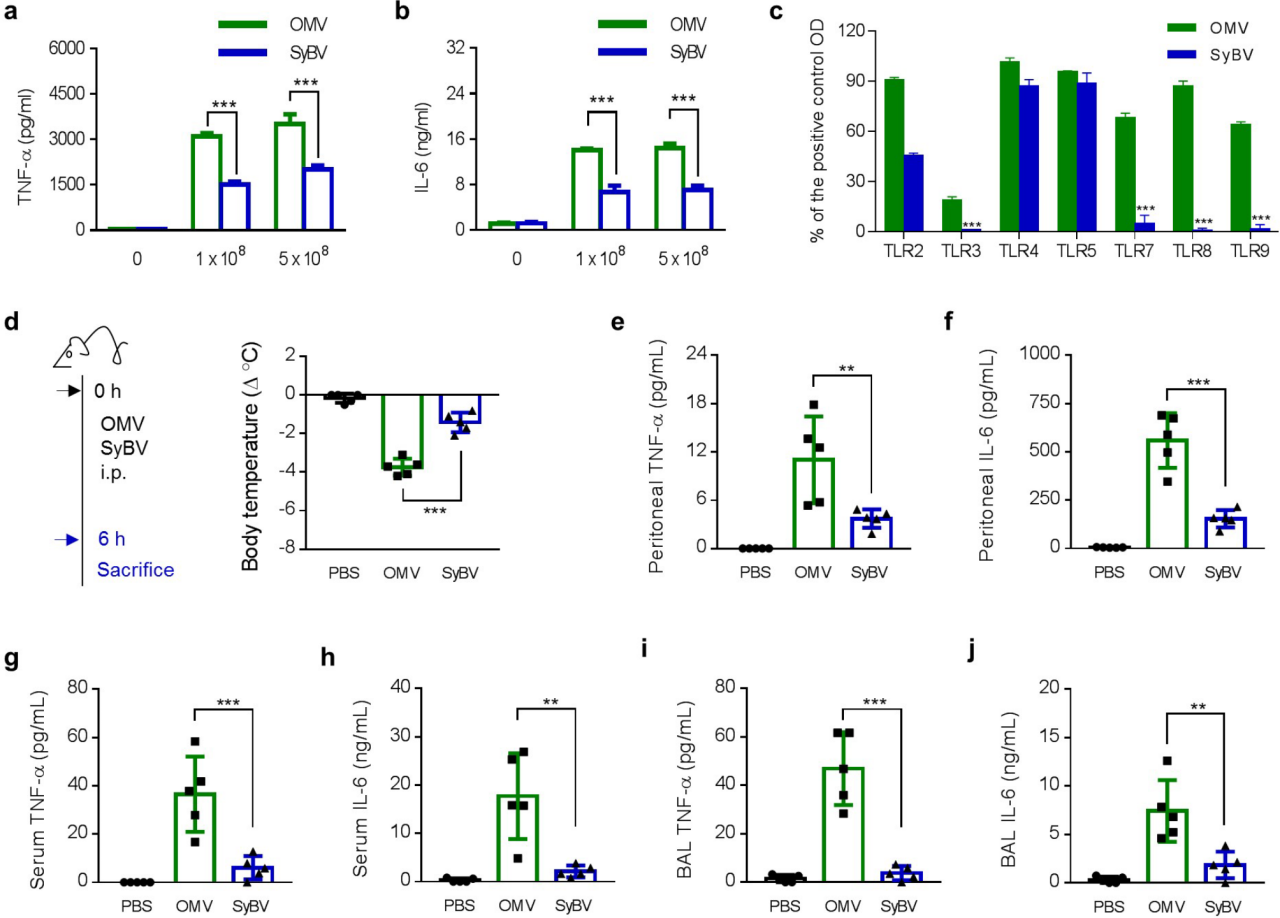
*P. aeruginosa* SyBV show reduced toxicity compared to natural OMV. **a, b** The secretion of the pro-inflammatory cytokines TNF-α (**a**) and IL-6 (**b**) from MH-S cells treated with two doses of OMV or SyBV for 24 h (*n* = 3 independent samples). **c** The relative contribution of *P. aeruginosa* OMV and SyBV to TLR signaling. OMV or SyBV were treated to HEK-293 cell lines overexpressing TLR and NF-ĸB reporter genes for 18 h (*n* = 2 independent samples), and then optical density (OD) value was measured. Data were indicated as percentage of OD of cells treated with a positive TLR agonist. Two-way ANOVA with Tukey’s post-test was used. **d** Study design for examining the toxicity of SyBV in mice (left panel). The same dose of OMV or SyBV (5 × 10^9^) was intraperitoneally injected into mice, and body temperature was evaluated at 6 h (right panel; *n* = 5 mice per group). **e, f** The production of TNF-α (**e**) and IL-6 (**f**) in the peritoneal fluid at 6 h (*n* = 5 mice per group). **g, h** The concentration of TNF-α (**g**) and IL-6 (**h**) in serum at 6 h (*n* = 5 mice per group). **i, j** The concentration of TNF-α (**i**) and IL-6 (**j**) measured in BAL fluid (*n* = 5 mice per group). All data are presented as the mean ± s.e.m. ^**^P < 0.01, ^***^P < 0.001 by one-way ANOVA with Tukey’s post-test

To evaluate the inflammatory response induced by SyBV in vitro, we used the alveolar macrophage cell line MH-S, which is known to respond to bacteria via the release of pro-inflammatory cytokines [14]. The OMV triggered a substantial pro-inflammatory cytokine response, including the release of tumor necrosis factor (TNF)-α and interleukin (IL)-6. In contrast, SyBV induced significantly less TNF-α and IL-6 release in the alveolar macrophage cell line MH-S (Fig. 2a, b). However, there was no significant difference in the cellular uptake of OMV and SyBV (Additional file 1: Fig. S5). Also, to examine which TLR signaling is involved in the decreased toxicity of SyBV, we measured the activity of TLR on recombinant HEK-293 cell lines in vitro. As a result, TLR3, 7, 8 and 9 were rarely responsive to SyBV compared to natural OMV (Fig. 2c), which is parallel with our previous result showing that gene materials were almost removed in the SyBV (Fig. 1e).

Next, the in vivo inflammatory profile of the SyBV was assessed using a mouse sepsis model with natural OMV challenge [8]. Mice were intraperitoneally injected with OMV or SyBV from *P. aeruginosa*, after which various inflammatory cytokines were investigated in the peritoneal cavity and serum. OMV caused hypothermia, a characteristic feature of the mouse sepsis model [15], whereas no significant difference in body temperature was observed in the SyBV-treated group compared to the phosphate-buffered saline (PBS)-treated group (Fig. 2d).

Because intraperitoneal challenge with OMV is linked to acute inflammation in the peritoneum, we next examined the profile of pro-inflammatory cytokines in the peritoneal cavity. While OMV induced a profound cytokine response, there was no significant increase in TNF-α (Fig. 2e) or IL-6 (Fig. 2f) in the peritoneal cavity of the SyBV-treated mice. Regarding systemic inflammatory responses, SyBV-treated mice had no difference in systemic cytokine release in comparison with PBS-treated mice (Fig. 2g, h). Given that OMV can stimulate inflammation at sites distant from the site of infection, such as the lungs and heart [8, 9], we next investigated if SyBV could induce inflammatory effects in the lung by assessing BAL fluid cytokines. In line with the cytokine patterns observed in the peritoneal fluid and serum, SyBV did not induce significant TNF-α and IL-6 expression in the bronchoalveolar lavage (BAL) fluid, whereas OMV induced expression of these cytokines (Fig. 2i, j). Taken together, these results suggest that the SyBV induced a significantly reduced inflammatory cytokine responses compared to natural OMV.

### Immunization with ***P. aeruginosa*** SyBV provides protection against bacterial lung infection in mice

**Fig. 3:**
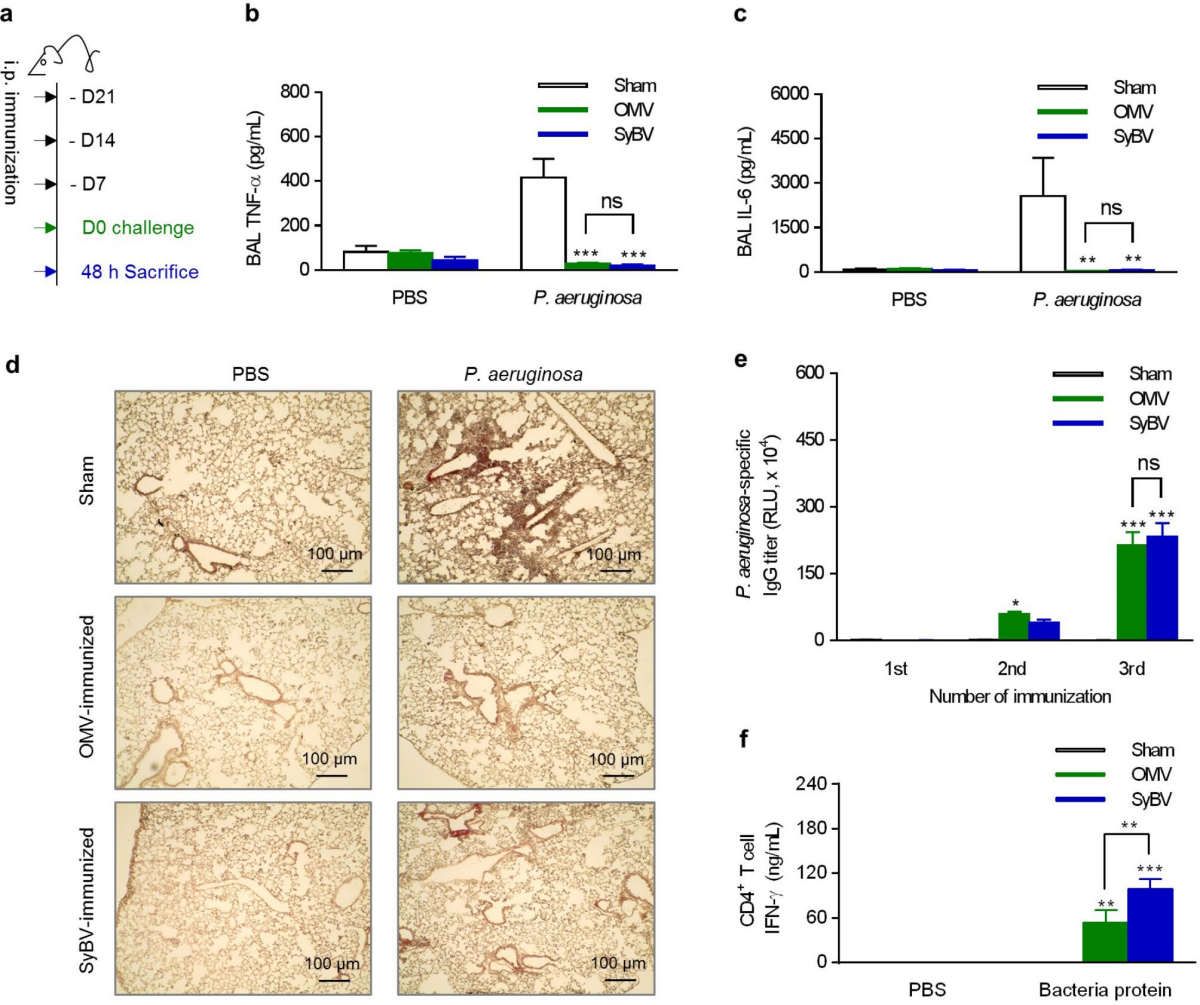
Immunization with *P. aeruginosa* SyBV inhibits the development of bacteria-induced pulmonary inflammation similar to natural OMV. **a** Study protocol for SyBV immunization for preventing bacteria-induced lung inflammation. **b, c** The level of TNF-α (**b**) and IL-6 (**c**) in BAL fluid was evaluated at 48 h after the last challenge (*n* = 5). **d** Representative lung histology images at 48 h after the last challenge (*n* = 5 with 10 images collected for each, scale bars: 100 μm). **e***P. aeruginosa*-specific IgG titers after the last immunization with OMV or SyBV (*n* = 5). **f** The level of *P. aeruginosa*-specific CD4^+^ T-cell-derived IFN-γ after CD4^+^ T-cells were isolated from immunized spleens (three independent samples). All data are presented as the mean ± s.e.m. ^**^P < 0.01, ^***^P < 0.001; ns, not significant by two-way ANOVA with Tukey’s post-test versus the sham group

Next, we examined the potential of immunization with *P. aeruginosa* SyBV to protect against *P. aeruginosa* in a mouse model of lung disease. Mice were injected intraperitoneally with SyBV three times at weekly intervals followed by an intranasal challenge with a non-lethal dose of *P. aeruginosa* (4 × 10^8^ CFU) one week after the last immunization, and mice were sacrificed two days after the challenge (Fig. 3a). There was no change in body temperature during immunization with OMV or SyBV (Additional file 1: Fig. S6). As shown in Fig. 3b, c, there was a dramatic decrease in the BAL levels of TNF-α and IL-6 in SyBV-immunized mice, which was comparable to the OMV-immunized mice. Further, a significant reduction in the infiltration of BAL neutrophils was observed in both the SyBV and OMV-immunized groups (Additional file 1: Fig. S7a), and this was concomitant with low levels of the neutrophil chemoattractant KC (CXCL1) in the BAL of the SyBV and OMV-immunized groups compared to the sham-immunized group (Additional file 1: Fig. S7b). Histological analysis in SyBV and OMV-immunized mice showed reduced inflammatory cell infiltration after infection compared to the control group (Fig. 3d), which was consistent with the cytokine and immune cell profiles.

To address the adaptive immune response caused by immunization with SyBV, we next investigated the humoral response by measuring the levels of antibodies specific to bacterial antigens. Immunization with SyBV induced an increased level of the bacteria-specific IgG antibodies compared to the sham group (Fig. 3e). Given that the T-cell immune response is also suggested to contribute to controlling bacterial infection [16], we next evaluated the bacteria-specific T-cell responses by assessing the production of key cytokines from splenic T-cells. Antigen recall stimulation of isolated splenic CD4^+^ T cells with *P. aeruginosa* strongly induced the release of the Th1 cytokine IFN-γ in mice immunized with SyBV and OMV (Fig. 3f). However, there was no change in the level of the Th2 cytokine IL-4 in any of the groups (Additional file 1: Fig. S8), suggesting that immunization with SyBV induces a specific Th1-type immune response.

### Immunization with ***E. coli*** SyBV provides protection against bacterial sepsis

**Fig. 4:**
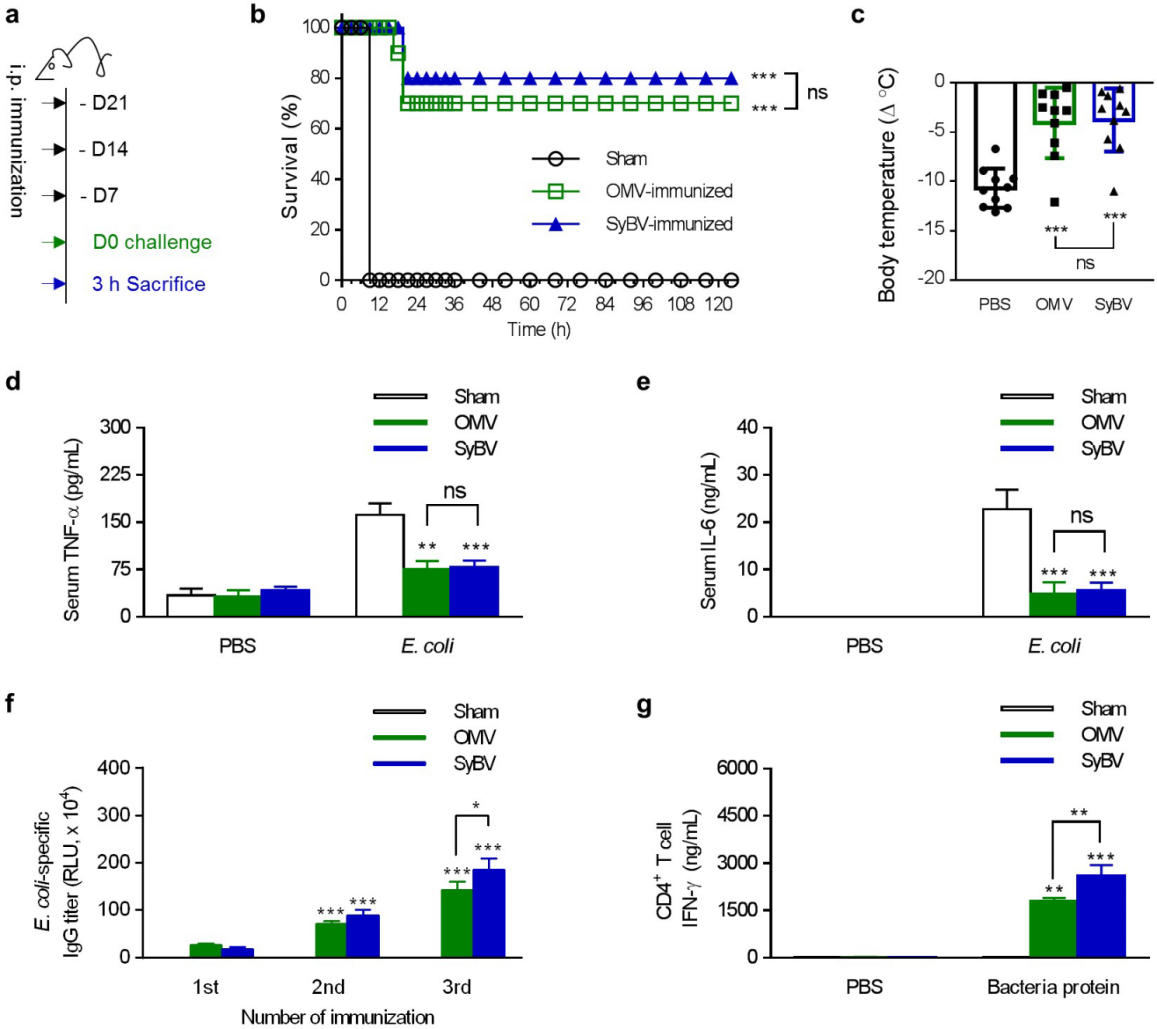
Immunization with *E. coli* SyBV protects against bacteria-induced lethality through suppression of systemic inflammation. **a** Study protocol for SyBV immunization and *E. coli* challenge for protecting against bacterial sepsis. **b** Survival curve measured for 5 days after intraperitoneal challenge with a lethal dose of *E. coli* (*n* = 10). **c** Body temperature was evaluated at 3 h following challenge with *E. coli* (*n* = 10). **d, e** The level of TNF-α (**d**) and IL-6 (**e**) in serum was quantified at 3 h after the last challenge (*n* = 5). **f***E. coli*-specific IgG titers after the last immunization with OMV or SyBV (*n* = 5). **g** The level of *E. coli*-specific CD4^+^ T-cell-derived IFN-γ after CD4^+^ T-cells were isolated from immunized spleens (three independent samples). All data are presented as the mean ± s.e.m. ^*^P < 0.05, ^**^P < 0.01, ^***^P < 0.001; ns, not significant by one-way or two-way ANOVA with Tukey’s post-test versus the sham group. For the survival curve, the Mantel–Cox log-rank test was used to compare with the sham group

We assessed the effect of immunization with *E. coli*-derived SyBV on protection against *E. coli*-induced sepsis in mice. Immunization with SyBV followed the protocol shown in Fig. 4a. Mice were actively immunized with SyBV once a week for three weeks and were then challenged with a lethal dose of *E. coli* one week after the last immunization. All mice in the control group were dead within 6 h after infection. However, over a five-day observation period, 80% of the mice in the SyBV-immunized group survived the challenge, and this was not significantly different compared to OMV-immunized mice (Fig. 4b). Further, the SyBV-immunized group did not show a decrease in body temperature, whereas the control group did (Fig. 4c). Moreover, no increase in the serum levels of TNF-α or IL-6 were observed in SyBV and OMV-immunized mice (Fig. 4d, e), showing that immunization with SyBV and OMV prevent *E. coli*-induced lethality via prevention of inflammatory responses.

Next, we evaluated the effect of SyBV immunization on the induction of *E. coli*-specific B-cell and T-cell responses. A rise in *E. coli*-reactive IgG antibody was observed after immunization with SyBV, whereas no specific IgG antibody was detected in the serum of sham mice (Fig. 4f). Further, antigen-specific IFN-γ production was observed in splenic CD4^+^ T-cells of the *E. coli* SyBV-immunized mice (Fig. 4g), which was similar to the previous finding in the *P. aeruginosa* SyBV-immunized mice (Fig. 3f). Also, to determine whether the observed protection is related to vesicular proteins on SyBV, we immunized mice with heat-inactivated SyBV and subsequently challenged the immunized mice with lethal dose of *E. coli*. As expected, there was a decreased survival rate (40%) in the heat-inactivated SyBV-immunized group (Additional file 1: Fig. S9), suggesting that vesicular proteins components should be important in the mechanism of immune protection. Collectively, these findings demonstrate that immunization with *E. coli* SyBV elicits protective immunity against an otherwise lethal *E. coli* challenge in mice, concomitant with the induction of antigen-specific IgG antibody and Th1 responses.

### Bacterial SyBV induce the proliferation and differentiation of adaptive immune cells in the spleen

**Fig. 5:**
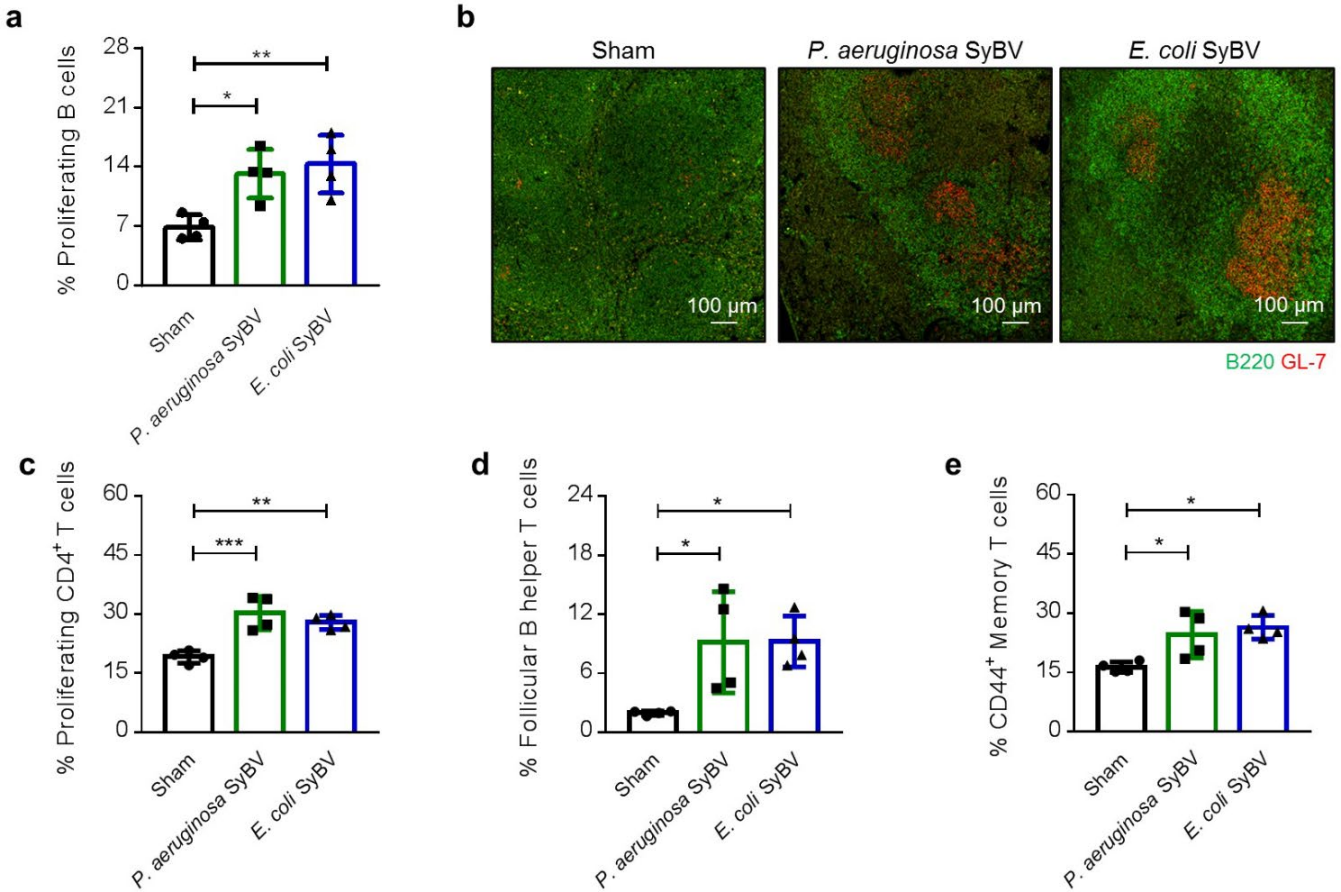
Immunization with bacterial SyBV induces proliferation and differentiation of germinal B-cells and T-cells in the spleen. **a** Mean percentages of proliferating B-cells observed in the spleens of mice immunized with *P. aeruginosa* or *E. coli* SyBV (*n* = 4). **b** Immunofluorescent staining of immunized mouse spleens for germinal center B-cells expressing B220/GL7. Scale bars, 100 μm. **c-e** Mean percentages of proliferating CD4^+^ T cells (**c**), follicular B-helper T-cells (**d**) and CD44^+^ memory T-cells (**e**) infiltrating the spleens of mice immunized with *P. aeruginosa* or *E. coli* SyBV (*n* = 4). Throughout, data are presented as the mean ± s.e.m. ^*^P < 0.05, ^**^P < 0.01, ^***^P < 0.001 by one-way ANOVA with Tukey’s post-test versus the sham group

Because B-cells are known to be activated by immunization with bacterial OMV [17], the nature of the B-cell response was explored in greater depth. Mice immunized with *P. aeruginosa* or *E. coli* SyBV showed a significant increase in the number of proliferating B-cells (Fig. 5a), and in particular a clear induction of splenic germinal centers and increased B-cell numbers were observed after immunization (Fig. 5b and Additional file 1: Fig. S10). We next assessed the population of CD4^+^ T-cells in the spleen, particularly follicular B-helper T-cells (CD4^+^CXCR5^+^Foxp3^–^) that are a specialized subset of CD4^+^ T-cells that play a critical role in protective immunity by helping B-cells to produce antibodies against pathogens [18]. As shown in Fig. 5c, immunization with SyBV induced a rapid increase in proliferating CD4^+^ T-cells in the spleen, including an increase in follicular B-helper T-cells (Fig. 5d) and memory T-cells (CD4^+^CD44^+^) (Fig. 5e), showing that bacterial SyBV immunization stimulates an extensive B-cell and T-cell response to protect against bacterial infection.

### Immunization with SyBV expressing SARS-CoV-2 spike protein elicits antibody and T-cell responses to SARS-CoV-2 in mice

**Fig. 6:**
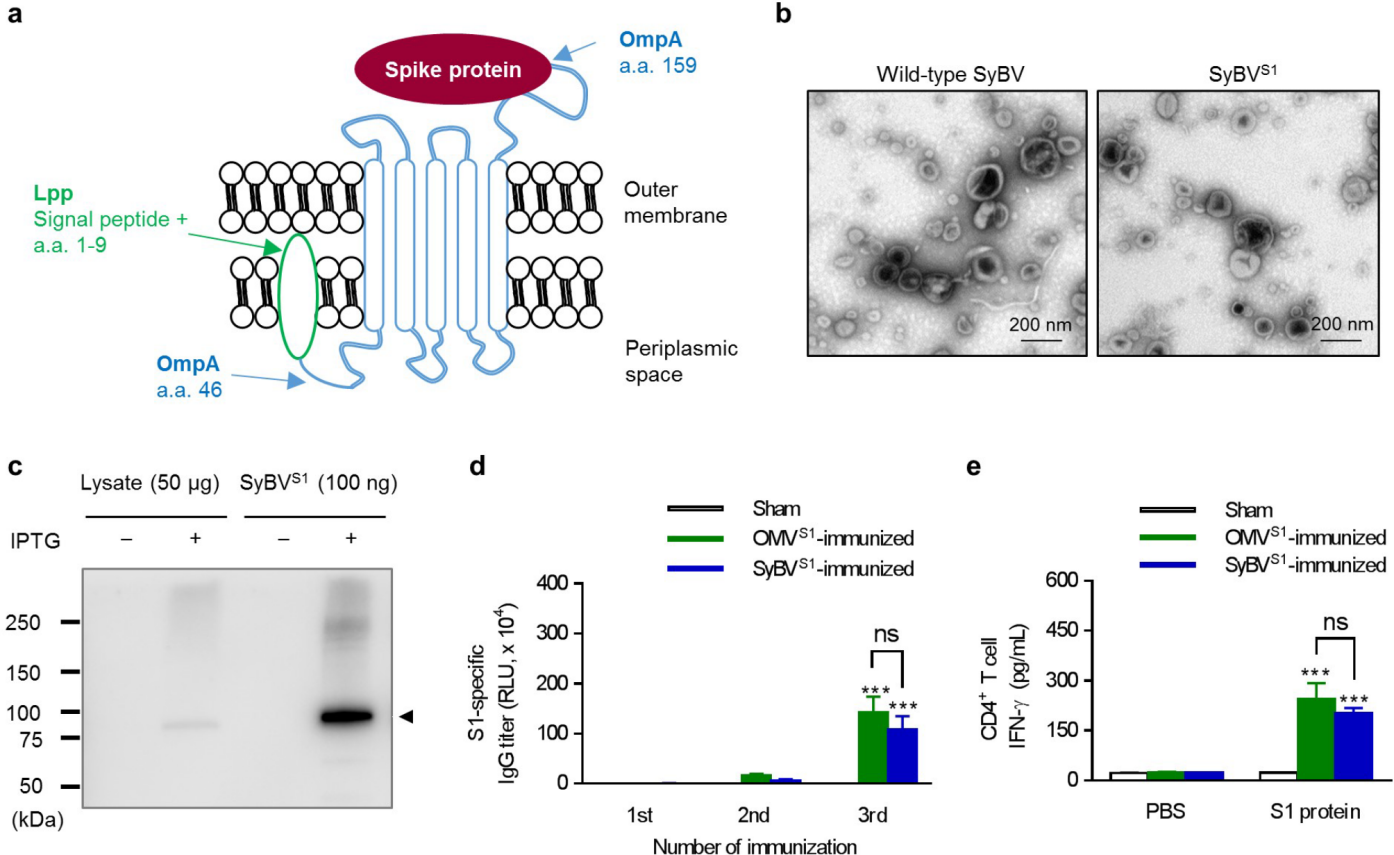
Bacterial SyBV overexpressing spike protein S1 stimulate virus-specific immunity through antibody production and T-cell activation. **a** Schematic representation of the structure of the chimeric Lpp-OmpA construct with SARS-CoV-2 spike protein S1 in the bacterial outer membrane. **b** TEM images of engineered SyBV overexpressing S1 (SyBV^S1^). Scale bars, 200 nm. **c** Western blot analysis of IPTG-induced bacterial lysates and SyBV^S1^ with anti-His Tag antibody. The filled triangle indicates the Lpp-OmpA-S1 complex. **d** S1-specific IgG titers after the last immunization with OMV^S1^ or SyBV^S1^ (*n* = 5). **e** The level of S1-specific CD4^+^ T-cell-derived IFN-γ after CD4^+^ T-cells were isolated from immunized spleens (three independent samples). All data are presented as the mean ± s.e.m. ^***^P < 0.001; ns, not significant by two-way ANOVA with Tukey’s post-test versus the sham group

We also explored whether a viral antigen can be presented by SyBV and, if so, whether the antigen-loaded SyBV can stimulate virus-specific immune responses. Spike protein S1, one of the active domains for SARS-CoV-2 entry, is a major target for the development of a SARS-CoV-2 vaccine [19]. Therefore, we used S1 as the model antigen protein to investigate the SyBV-mediated vaccination effect. To express S1 on the membrane of SyBV, we employed the Lpp-OmpA chimera platform, which has been shown to help display polypeptides such as beta-lactamase, cellulase, and scFv antibodies on the cell membrane [20]. A Lpp-OmpA-S1 fusion plasmid was constructed and transformed into bacteria to overexpress S1 on the bacterial surface (Fig. 6a). The S1-loaded SyBV (SyBV^S1^) were manufactured, and TEM image of the vesicles revealed similar morphological characteristics as wild-type SyBV (Fig. 6b). The presence of the S1 protein on the SyBV was confirmed by Western blotting analysis, indicating a greater enrichment of S1 on SyBV than in whole-cell lysate (Fig. 6c).

We assessed whether S1 antigen delivered by SyBV can induce adaptive immune responses. Mice were immunized with SyBV^S1^ three times at one-week intervals. All SyBV^1^-immunized mice developed high levels of S1-specific IgG antibodies in their sera (Fig. 6d), and S1-specific induction of a Th1 cytokine IFN-γ response was observed in the spleen from SyBV^1^-immunized mice (Fig. 6e). No appreciable level of the Th2 cytokine IL-4 was observed (Additional file 1: Fig. S11). To compare the immunogenic activity of SyBV^S1^ with S1-loaded OMV (OMV^S1^), OMV^S1^ were first purified from the supernatants of an engineered bacterial culture and S1 expression was confirmed by Western blotting (Additional file 1: Fig. S12). Immunization with OMV^S1^ also showed a significant increase in S1-specific IgG antibody and splenic IFN-γ secretion similar to immunization with SyBV^S1^ (Fig. 6d, e). Taken together, these results suggest that the SyBV platform can efficiently deliver viral S1 protein and that the engineered vesicles provoke S1-specific antibodies and Th1-type responses.

## Discussion

In this work we show that SyBV can function as a novel vaccine platform, combining both adjuvant function and antigen presentation concomitantly in the same structure. Briefly, outer membranes from bacteria are isolated through a process that includes enzymatic treatments and ionic stress, which removes much of the cytosolic components. The SyBV are then manufactured by exposing the isolated membranes to ultrasonic energy. Immunization with SyBV from *P. aeruginosa* resulted in bacteria-specific immunity and protection against infection. Similarly, making the SyBV from *E. coli* resulted in immunity against the same bacteria, including protection against a fatal bacterial challenge. We further explored the possibility of engineering the bacterial membrane to express virus protein, and as a model we expressed the SARS-CoV-2 S1 protein, which also resulted in virus-protein specific immune responses.

Our group previously proposed an alternative strategy for producing artificial vesicles directly from *E. coli* outer membranes, and we verified that the vesicles were non-toxic and had an immunotherapeutic potential against cancer progression [11]. In the current project, we exploited this technology by making SyBV from *P. aeruginosa*, which is an important pathogen in chronic obstructive pulmonary disease and cystic fibrosis and is characterized by increased antibiotic resistance [21]. *P. aeruginosa* SyBV could be generated with increased purity compared to the naturally released OMV, as illustrated by TEM (Fig. 1b) and by reduced nucleotide content (Fig. 1e). Moreover, the protein composition of SyBV revealed fewer cytosolic proteins compared to natural OMV (Fig. 1h), thus illustrating the enrichment of the outer membrane. These data suggest that our technology for manufacturing SyBV may be universally applied to any Gram-negative bacteria and is an effective way to resolve the impurity problem with OMV.

In recent years, natural OMV have been tested as a form of vaccine against bacterial infection due to their immunotherapeutic potential, their nanosized structure suitable for crossing cell membranes, and their flexibility in carrying heterologous antigens [22, 23]. However, most OMV-based vaccines are still in the non-clinical testing stage, although the Meningococcus OMV vaccine Bexsero® is clinically available. One of the critical issues for OMV-based vaccines is the often severe side effects they can induce, both locally and systemically. Indeed, it has been confirmed that bacterial OMV cause systemic inflammation through an excessive cytokine storm, disseminated intravascular coagulation, and multiple organ dysfunctions, including in the heart [8, 9, 24]. Especially, *P. aeruginosa* has been known to be secrete toxic OMV that cause severe lung inflammation [10]. We here chose *P. aeruginosa* to develop much safer vaccine for rather than OMV. We here confirm that the SyBV showed reduced pro-inflammatory cytokine response in alveolar macrophages and in mice compared to OMV (Fig. 2), suggesting that the SyBV are safer for vaccine purposes. Also, our process for SyBV production was directly applied to bacterial membranes, enabling high production of vesicles as shown in Fig. 1c. Thus, SyBV-based vaccines can be considered a cost-effective platform compared to other traditional adjuvants like alum [25]. Regarding the uptake of bacterial vesicles by immune cells, it has been shown that the vesicles can be easily delivered to macrophages through various internalization routes like receptor-mediated endocytosis and fusion [26]. Here we did not compare the internalization patterns of SyBV and OMV; however, it is expected that they have analogous potencies to be transferred to target cells because of their similar nano-scaled size with outer membrane surface proteins.

It has been reported that immunization with *P. aeruginosa*-derived OMV can give protective immunity against *P. aeruginosa* infection through diminished cytokine production and cell infiltration into lung tissue [7], which was also observed in our OMV-immunized group (Fig. 3). Then, we asked whether the detoxified SyBV maintain their immunizing capacity compared to the naturally released OMV. Our data shown in Fig. 3 clearly indicate that pre-immunization with SyBV could protect against an inflammatory response and could elicit evident immunity against *P. aeruginosa* infection, which was comparable to immunization with the non-detoxified OMV. However, it is still unclear which protein components contribute to the immune protection and which molecules are important for the adjuvant function. Although it is difficult to identify specific proteins related to immunogenicity due to the large number of proteins in vesicles, there have been several reports that some outer membrane proteins such as OprF and OprG present on OMV are able to induce specific protective immunity [27–30]. Gilleland et al. reported that rats immunized against OprF showed significant protection against the onset of severe pulmonary lesion [28]. Also, active immunization against OprF could induce specific humoral responses in clinical trials, and no safety concerns were reported [29]. Interestingly, these outer membrane proteins were also found in the SyBV proteome (Additional file 1: Table S1), implying that a specific subset of outer membrane proteins might be the main components concerning SyBV-induced protective immunity. OMV have been known to exhibit more protective potential compared to single protein [31]. For instance, *Neisseria meningitidis*-derived OMV expressing a specific porin protein induced much bacterial antibodies than the purified porin protein [32]. In the same context, we postulate that the SyBV immunization may be more efficient than vaccination with any single molecule because a broader immune response against multiple bacterial proteins and epitopes will be produced.

Various Gram-negative bacteria-derived OMV have demonstrated extensive protection in mouse models of sepsis [33–35]. In particular, *E. coli*, which is a general causative pathogen of sepsis, has been shown to release OMV containing highly immunogenic outer membrane proteins such as OmpA, OmpC, and OmpF [36]. Although *E. coli* OMV have been shown to prevent bacteria-induced lethality and systemic inflammation when used to immunized mice [33], no one has yet been able to resolve the safety concerns of *E. coli* OMV-based vaccines. As mentioned above, we previously showed that *E. coli* is a suitable strain to efficiently generate non-toxic SyBV by various enzymes and sonication, and the vesicles elicited significant tumor regression in melanoma-bearing mice [11]. In the current study, the immunoprotective activity of *E. coli* SyBV was also demonstrated in an *E. coli*-induced sepsis model. The active immunization with *E. coli* SyBV conferred successful protection and a potent antigen-specific adaptive immune response (Fig. 4), suggesting that SyBV might be versatile and safe adjuvants for various disease models such as cancer and infection.

Most available adjuvants like alum are potent in inducing a profound antibody response [37]. Although immunoglobulins have been shown to confer protection against infectious agents, humoral responses alone may not be sufficient to protect against infection [38, 39], and it is considered preferable to stimulate concomitant strong B-cell and T-cell immunity in order to achieve clinical success. Thus, we investigated whether SyBV-based vaccines could also activate cellular T-cell immunity. Active immunization with both *P. aeruginosa*-derived and *E. coli*-derived SyBV stimulated antigen-specific T-cell responses together with a dramatic increase in antibody titer (Figs. 3 and 4), suggesting that our vaccine platform is a good strategy for augmenting vaccine efficacy through a balanced humoral and cellular response. Also, the level of the Th1 cytokine IFN-γ released by CD4^+^ T cells in the spleen was boosted after immunization with SyBV (Figs. 3f and 4 g), which was also observed by Kim et al. using engineered bacterial vesicles to protect against sepsis [40]. A growing number of studies have shown that OMV-based vaccines induce both humoral and cellular immunity for complete protection [33, 41], but the relative importance of humoral versus cellular immunity is not well determined in the protective mechanism of bacterial vesicles. We here found that both B-cells and T-cells were activated in the spleen of SyBV-immunized mice (Fig. 5); however, further investigations in gene-knockout mouse studies are needed to determine which responses between B-cell-derived humoral and T-cell-derived cellular immune defenses are relatively dominant in SyBV-induced adaptive immunity. Also, we cannot ignore the possible contribution of innate immune cells and non-T-cells such as natural killer cells and dendritic cells, thus the relative significance of other immune cells in SyBV-based immunity needs to be clarified.

OMV have been suggested to be a flexible vaccine platform because exogenous heterologous proteins can be easily engineered into the OMV membrane [22]. The major benefit of engineered bacteria-derived OMV is that expressed antigens in OMV maintain their conformation and their potency in targeting antigen-specific immune responses. Different approaches have been applied to display proteins of interest on the vesicular membrane [42]. For example, transmembrane protein cytolysin A and hemoglobin protease-based autotransporter platforms have been used to display recombinant proteins on the vesicles [43, 44]. Also, glycosylphosphatidylinositol (GPI)-anchoring tool has been used for lipid membrane engineering to carry out a specific protein [45].

In this study, we used the Lpp-OmpA chimeric system developed by Georgiou et al. to display the SARS-CoV-2 S1 protein because this anchoring strategy has been shown to express both enzymes and antibody fragments [46, 47]. Although the intrinsic adjuvant effect of OMV in bacterial infection is well understood [48, 49], it has been poorly studied in viral diseases. However, *Neisseria meningitidis*-derived OMV have been shown to efficiently induce humoral and cellular responses against HIV-1 virus [50], and Watkins et al. have demonstrated that OMV constructed to display influenza A virus matrix 2 protein conferred 100% survival against a lethal challenge with influenza virus A [51]. In this work we delivered SARS-CoV-2 S1 protein via non-toxic SyBV to elicit viral antigen-specific immunity. We found that immunization with the recombinant SyBV induced high concentrations of S1-specific IgG antibodies (Fig. 6d). Also, the level of the Th1 cytokine IFN-γ released by CD4^+^ T-cells in the spleen was enhanced after immunization with SyBV^S1^ (Fig. 6e), strongly suggesting that SyBV can be a universal vaccine platform for various infectious diseases, including viruses.

## Conclusions

Our study shows for the first time the prophylactic effect of a SyBV-based therapeutic vaccine against mouse pneumonia and sepsis, and it demonstrates the contribution of humoral and cellular immune responses to bacterial antigen-specific adaptive immunity. Moreover, our results show that SyBV could be engineered to efficiently deliver exogenous viral antigens, which could be utilized to develop vaccines against viruses, such as SARS-CoV-2. We confirm that SyBV could be generated from various Gram-negative bacteria and even engineered strain, thus there is possible future developments of potentially safer and more potent vaccines by using LPS-mutant or specific immunogenic protein-overexpressing strain.

Even though SyBV appear to be a promising platform for developing multiple types of immunoregulatory vaccines, there are still several hurdles that needs to be considered. First, further studies are needed to determine in more detail which components in the SyBV convey the adjuvant effect and which molecules are important for the specific immunity. Second, in order to translate the work to clinical use, an efficient GMP manufacturing process needs to be developed that eliminates the need for relatively low-volume steps such as ultracentrifugation. Third, it may be important to consider the difference in glycosylation of bacterially expressed viral proteins because bacterial membrane proteins are less glycosylated compared to proteins produced by eukaryotic cells, which may result in slightly different antigen structures.

## Methods

### Animals

Wild-type mice in the C57BL/6 genetic background (6 weeks old) were purchased from Charles River. The mice were maintained at the Experimental Biomedicine facility at the University of Gothenburg, Sweden. The study was approved by the local Animal Ethics Committee in Gothenburg, Sweden (Dnr 5.8.18–03598/2019), and was carried out according to institutional animal use and care guidelines.

### Bacterial strains and cell culture

*P. aeruginosa* PAO1 (ATCC, Manassas, VA) and *E. coli* BL21 (DE3) (Thermo Fisher Scientific, Waltham, MA) were cultured in Luria-Bertani broth at 37 °C. MH-S (ATCC), a murine alveolar macrophage cell line, was maintained in RPMI 1640 medium (HyClone, Logan, UT) containing 10% FBS, 2 mM L-glutamine, 100 U/mL penicillin, and 100 µg/mL streptomycin. The cells were cultured at 37 °C in an atmosphere of 5% CO_2_.

### Isolation of natural OMV

Bacterial cultures were pelleted at 6,000 × *g* at 4 °C for 20 min, and the supernatants were applied to 0.45 μm vacuum filters to remove cell debris. The filtrate was concentrated with a Vivaflow 200 module equipped with a 100 kDa cut-off membrane (Sartorius, Goettingen, Germany). The concentrated solution was ultracentrifuged at 150,000 × *g* at 4 °C for 3 h to pellet the OMV, and the pelleted OMV were resuspended with PBS.

### Preparation of SyBV

To collect bacterial outer membranes, the bacterial cultures were first pelleted and resuspended in 20 mM Tris-HCl (pH 8.0) with lysozyme (600 µg/g cells) and 0.1 M EDTA (0.2 mL/g cells). The resulting spheroplasts were sonicated, and total membranes were obtained by centrifuging at 40,000 × *g* for 1 h at 4 °C (Fig. 1a). The outer membranes were isolated by incubation with 0.5% Sarkosyl (Sigma Aldrich, St. Louis, MO) followed by centrifuging at 40,000 × *g* for 1 h at 4 °C. The outer membrane pellets were treated with high pH solution (200 mM Na_2_CO_3_ (pH 11)) to disrupt the membrane integrity. The open membranes were resuspended in 4 mL of 50% iodixanol (Axis-Shield PoC AS, Oslo, Norway) and applied onto a step gradient (4 mL of 30% iodixanol and 2 mL of 10% iodixanol) in a 14 mL ultracentrifuge tube (Beckman Coulter, Brea, CA). The tube was ultracentrifuged at 100,000 × *g* for 2 h at 4 °C to collect vesicles between the 10% and 30% iodixanol layer. Finally, the vesicles were mildly sonicated and considered as SyBV.

### TEM

OMV and SyBV were analyzed by negative-stain TEM. The vesicles were applied onto glow-discharged formvar carbon-coated 200-mesh copper grids (Electron Microscopy Sciences, Hatfield, PA). After washing with water, the vesicles were fixed with 2.5% glutaraldehyde dissolved in PBS, followed by staining with 2% uranyl acetate for 1.5 min. Negative-stained bacterial vesicles were visualized using a LEO 912AB Omega electron microscope (Carl Zeiss SMT, Oberkochen, Germany) at 120 kV with a Veleta CCD camera (Olympus-SiS, Stuttgart, Germany).

### Nanoparticle tracking analysis

OMV and SyBV (10 µg/mL) were measured using ZetaView® PMX 120 (Particle Metrix GmbH, Meerbuch, Germany). Measurements were done in triplicate, and each individual data point was obtained from two stationary layers with five measurements in each layer. The sensitivity of the camera was set to 70 for all measurements. Data were interpreted using ZetaView analysis software version 8.2.30.1 with a minimum size of 10, a maximum size of 1000, and a minimum brightness of 30.

### RNA analysis

RNA from bacterial vesicles was purified with the miRCURY™ RNA isolation kit for biofluids (Exiqon, Vedbaek, Denmark) according to the manufacturer’s protocol. One microliter of isolated RNA was examined for its quality, yield, and nucleotide length by capillary electrophoresis using an Agilent RNA 6000 Nanochip on an Agilent 2100 Bioanalyzer® (Agilent Technologies GmbH, Berlin, Germany).

### LC-MS/MS analysis

Two biological replicates of OMV and SyBV (30 µg of both) were digested with trypsin using the filter-aided sample preparation (FASP) method and C18 spin columns according to the manufacturer’s instructions [52]. All fractions were dried on a Speedvac and reconstituted in 3% acetonitrile and 0.2% formic acid and examined on an Orbitrap Fusion Tribrid mass spectrometer interfaced with an Easy-nLC 1200 (Thermo Fisher Scientific). Peptides were captured on an Acclaim Pepmap 100 C18 trap column (100 μm × 2 cm, particle size 5 μm; Thermo Fischer Scientific) and separated on an in-house packed C18 analytical column (75 μm × 30 cm, particle size 3 μm) using a gradient from 5.6 to 80% acetonitrile in 0.2% formic acid over 90 min at a flow of 300 nL/min. Precursor ion mass spectra were monitored at 120,000 resolution, and the most intense precursor ions were fragmented using HCD at a collision energy setting of 30. The MS/MS spectra were recorded at 30,000 resolution with a maximum injection time of 110 ms and an isolation window of 1.2 Da. Charge states 2 to 7 were selected for fragmentation, and dynamic exclusion was set to 20 s with 10 ppm tolerance.

### Database search

Data were analyzed with Proteome Discoverer version 1.4 (Thermo Fisher Scientific). The database search was performed against the Swissprot *P. aeruginosa* database. Mascot 2.5.1 (Matrix Science, London, UK) was used as the search engine with a precursor mass tolerance of 5 ppm and fragment mass tolerance of 0.5 Da, and one missed cleavage was recognized, mono-oxidation on methionine was set as a variable modification, and methylthiolation on cysteine was set as a fixed modification. Percolator was used for the validation of the identification results with the strict target false discovery rate of 1%, and proteins were only accepted when identified in all replicates. Gene ontology analysis was performed using the Funrich analysis tool, and principal component analysis and hierarchical cluster analysis were performed with the ClustVis software. The mass spectrometry data has been deposited to the ProteomeXchange Consortium via the PRIDE partner repository with the dataset identifier PXD033690.

### Immunotoxicity assay

The immunotoxicity of the OMV and SyBV was compared with a cytokine release assay in MH-S cells. The cells were applied onto 24-well plates and two concentrations of OMV or SyBV were added, and pro-inflammatory cytokine release was measured 24 h later. The secretion of TNF-α and IL-6 in the supernatants was quantified by a DuoSet ELISA Development kit (R&D Systems, Minneapolis, MN). For the in vivo safety assay, mice were intraperitoneally injected with the same number of OMV or SyBV (5 × 10^9^) and then sacrificed after 6 h following anesthetization with intraperitoneal injection of xylazine chloride (10 mg/kg; Bayer, Gothenburg, Sweden) and ketamine hydrochloride (100 mg/kg; Pfizer AB, Kent, UK). Rectal temperature was measured with a thermometer (Bioseb, Chaville, France). Blood was acquired by cardiac puncture and peritoneal fluid and BAL fluid were acquired, and after centrifugation the supernatants were maintained at − 80 °C for cytokine analysis.

### TLR screening assay

Engineered HEK-293 cell lines expressing different murine TLR were purchased by InvivoGen (Toulouse, France). In case of TLR2, only single receptor was tested without heterodimer formation with TLR1 or TLR6. The receptors were linked with a reporter gene which is a secreted alkaline phosphatase. The activation of the reporter gene was mediated by a NF-ĸB inducible promoter, and then TLR activation results were determined as optical density values after 18 h stimulation of the cells with OMV or SyBV. As a positive control for activation of murine, the following ligands were used: TLR2 (PAM2), TLR3 (Poly I:C), TLR4 (LPS), TLR5 (Flagellin), TLR7 (R848), TLR8 (TL8-506), and TLR9 (ODN 1826).

### Cell uptake experiment

OMV or SyBV were stained with DiO (Molecular Probes, Eugene, OR) for 1 h at 37 °C. MH-S cells labelled with Cellmask Deep Red (Thermo Fisher Scientific, Waltham, MA) were incubated with the DiO-labelled vesicles for 6 h. Flow cytometry was analyzed using a BD FACSVerse Flow Cytometer running BD FACSuit Software (BD Biosciences, San Jose, CA) and FlowJo Software (Tree Star Inc., Ashland, OR).

### Immunization protocol and induction of pulmonary inflammation

Mice were intraperitoneally injected with 5 × 10^9^*P. aeruginosa*-derived OMV or SyBV once a week for three weeks. For *P. aeruginosa*-induced lung inflammation, mice were intranasally challenged with 4 × 10^8^ CFU of *P. aeruginosa* followed by collection of BAL fluid and lungs after 48 h. For lung histology, lungs were fixed with 4% paraformaldehyde, sectioned at 4 μm, and stained with hematoxylin and eosin. The images were acquired using the EVOS XL Core Imaging System (Life Technologies, Bothell, WA).

### Immunization protocol for sepsis

Mice were intraperitoneally immunized with 5 × 10^9^*E. coli*-derived OMV or SyBV three times every week. For *E. coli*-induced sepsis, immunized mice were intraperitoneally injected with *E. coli* (1 × 10^8^ CFU), and survival was monitored every 3 or 12 h for 5 days. Rectal temperature and serum cytokines were measured 3 h after challenge with *E. coli*. For heat inactivation of SyBV, the vesicles were incubated for 20 min at 100 °C. And then survival rate of mice immunized with heat-inactivated SyBV (5 × 10^9^) was determined for 5 days following challenge with lethal dose of *E. coli*.

### Measurement of antibody titers against bacterial proteins

Serum samples were obtained from mice 3 days after each immunization and assayed for IgG antibodies specific for bacterial proteins by ELISA. Briefly, the mouse serum was diluted 1:500 in 1% BSA/PBS and applied to 96-well plates coated with 200 ng bacterial lysates. After incubation for 2 h, the IgG antibody levels were quantified with a peroxidase-conjugated anti-mouse IgG antibody followed by a luminescent substrate (Thermo Fisher Scientific).

### Cytokine release by splenic T-cells

CD4^+^ T-cells were obtained from mouse spleens following immunization using a cell isolation kit (Miltenyi Biotec, Bergish Gladbach, Germany) according to the manufacturer’s instructions. The CD4^+^ T-cells (5 × 10^5^) were incubated for 72 h with 1 µg/mL of bacterial proteins, followed by measurement of IFN-γ and IL-4 in the supernatants using a DuoSet ELISA Development kit (R&D Systems).

### Flow cytometry analysis

Mouse spleens were removed upon sacrifice after immunization, and single cell suspensions were prepared. Viable cells were blocked for non-specific staining with 2.4G2 (anti-Fc-receptor) and stained with the following antibodies: anti-CD4-APC-H7 (GK1.5), anti-CD19-BUV395 (1D3), anti CD62L-BV605 (MEL-14), anti-CD44-BV786 (IM7), anti-CXCR5-BV605 (2G8), anti-Rorgt-PECF594 (Q31-378), anti-ki67-V450 (B56), and anti-BCL6-AF488 (K112-91) obtained from BD Biosciences (San Jose, CA); anti-TCRb-PeCy7 (H57-597), anti-MHCII-AF700 (M5/114.15.2), anti-PD1-BV711 (29 F.1A12), and anti-Foxp3-AF647 (150D) obtained from Biolegend (San Diego, CA); and anti-GL7-PE (GL-7) obtained from eBioscience (San Diego, CA). To exclude dead cells, Live/dead Aqua (Thermo Fisher Scientific) was used to exclude positive cells from the analysis. Intracellular staining was performed using the FOXP3 transcription factor staining kit (eBioscience) according to the manufacturer’s instructions. Events were collected and analyzed using an LSR-II or LSR Fortessa-X20 (BD Biosciences) and FlowJo software (Treestar Inc., Ashland, Oregon).

### Immunohistochemistry

Mouse spleens were obtained upon sacrifice after immunization and fixed in 4% paraformaldehyde and 10% sucrose for 1 h and then transferred to 30% sucrose solution for overnight incubation at 4 °C. The spleens were embedded in TissueTek OCT compound and snap frozen in liquid nitrogen. Frozen Sects. (7–9 μm thick) were fixed in 100% acetone and blocked with 5% normal horse serum in PBS for 15 min. The antibodies used to stain sections were anti-mouse B220-FITC (clone: RA3-6B2; BD Biosciences, San Jose, CA) and GL-7-biotin (clone: GL7; eBioscience) followed by Streptavidin-Alexa Flour 594 (Thermo Fisher Scientific). Microscopy was performed with a confocal Zeiss LSM 700 inverted system and LSM software (Carl Zeiss, Oberkochen, Germany).

### Display of spike protein S1 on the bacterial surface

*E. coli* BL21 (DE3) was used to express recombinant fusion proteins. Plasmid pET-28a(+) contained a fusion of the signal sequence and the first nine amino acids of Lpp, the sequence for five outer membrane-spanning domains of OmpA, the full sequence for the SARS-CoV-2 spike protein S1, and a His tag. For S1 overexpression, transformed bacterial cultures were inoculated at a ratio of 1/50 from overnight cultures and grown in a large volume of medium supplemented with 50 µg/mL kanamycin. When cultures reached an optical density of OD600 = 0.6, protein expression was induced by the addition of 0.1 mM IPTG (Thermo Fisher Scientific).

### SDS-PAGE

Bacterial whole-cell lysates and SyBV overexpressing S1 were separated by 10% SDS-PAGE and transferred to a polyvinylidene difluoride membrane. The blocked membrane was then incubated with anti-His tag antibody (Thermo Fisher Scientific). After incubation with horseradish peroxidase-conjugated secondary antibody, the immunoreactive bands were detected with a chemiluminescent substrate.

### Immunization with vesicles expressing S1

Mice were intraperitoneally injected with 5 × 10^9^ OMV or SyBV overexpressing S1 three times every week. Serum samples were taken from mice 3 days after each immunization to measure the antibody titer against recombinant S1 proteins (Arigo Biolaboratories, Hsinchu City, Taiwan). For splenic cytokines, isolated CD4^+^ T-cells (5 × 10^5^) were incubated for 72 h with 1 µg/mL of S1 proteins followed by measurement of cytokines in the supernatants using a DuoSet ELISA Development kit (R&D Systems).

### Statistical analysis

Data analysis was performed using GraphPad Prism 7. Results are shown as means and standard errors of the mean. Unpaired two-tailed Student’s *t*-test was performed to compare two groups. One-way ANOVA followed by Tukey’s multiple comparison test was used to evaluate the difference between multiple groups with one independent variable, and two-way ANOVA was applied to compare multiple groups with two independent variables followed by Tukey’s multiple comparison test. Statistical significance for the survival curve was evaluated by the Mantel–Cox log-rank test. *P* < 0.05 was considered to be statistically significant.

## Electronic supplementary material

Below is the link to the electronic supplementary material.

Supplementary Material 1

**Additional file 1: figure S1:** Schematic overview of OMV and SyBV isolation from cultured bacteria. **Figure S2.** Characterization of RNA isolated from OMV and SyBV. Representative electropherograms of RNA molecules isolated from SyBV in comparison to those from OMV. The filled triangle indicates internal marker. **Figure S3.** Quantification of DNA isolated from OMV and SyBV (*n* = 3 independent samples). Data are presented as the mean ± s.e.m. ns, not significant by unpaired two-tailed Student’s *t*-test. **Figure S4.** Comparison of protein composition between *P. aeruginosa* OMV and SyBV. (**a**) Principle component analysis of OMV and SyBV proteome. Two biological replicates per sample and three technical replicates for each biological replicate. (**b**) Partial view of heatmap of the hierarchical clustering based on the relative proteins abundance of OMV and SyBV proteome. Color code shows the normalized median abundance of proteins belonging to the category (red, most abundant; blue, least abundant). **Figure S5.** SyBV can be efficiently taken up by macrophages comparable to OMV. DiO-labelled vesicles (1 × 10^9^) were incubated with MH-S cells for 6 h. And, the uptake of SyBV by cells was compared with OMV by flow cytometry, and the results are shown as the percentage of DiO-positive cells (*n* = 3). Data are presented as the mean ± SEM. ^***^P < 0.001; ns, not significant, by one-way ANOVA with Tukey’s post test. **Figure S6.** Measurement of body temperature during immunization with *P. aeruginosa* OMV or SyBV. The temperature of mice was investigated at 24 h after each immunization (*n* = 5). Data are presented as mean ± s.e.m. ns, not significant; by one-way ANOVA with Tukey’s post test versus the sham group. **Figure S7.** Differential cell count and chemokine level in BAL fluid of mice immunized with *P. aeruginosa* OMV or SyBV. (**a**) The effect of OMV and SyBV on the numbers of BAL cells (macrophage, neutrophil and lymphocyte) was investigated at 48 h after the last challenge (*n* = 5). (**b**) The level of neutrophil-chemoattractant chemokine (KC) in BAL fluid was evaluated at 48 h after the last challenge (*n* = 5). Data are presented as mean ± s.e.m. ^***^P < 0.001; ns, not significant; by two-way ANOVA with Tukey’s post test versus the sham group. **Figure S8.** The level of *P. aeruginosa*-specific CD4^+^ T-cell-derived IL-4 after CD4^+^ T-cells were isolated from immunized spleens (three independent samples). All data are presented as the mean ± s.e.m. ns, not significant by two-way ANOVA with Tukey’s post-test versus the sham group. **Figure S9.** Survival curve of mice immunized with heat-inactivated SyBV from *E. coli*. The result was monitored for 5 days after intraperitoneal challenge with lethal dose of *E. coli* (*n* = 10). For survival curve, log-rank (Mantel-Cox) test was used to compare with sham group (*P < 0.05). **Figure S10.** Quantification of germinal center B cells. Graph shows cell counts per germinal center cross-section in the mice spleens immunized with *P. aeruginosa* or *E. coli* SyBV. Data are presented as mean ± s.e.m. ***P < 0.001 by one-way ANOVA with Tukey’s post test. **Figure S11.** The level of S1-specific CD4^+^ T-cell-derived IL-4 after CD4^+^ T-cells were isolated from immunized spleens (three independent samples). All data are presented as the mean ± s.e.m. ns, not significant by two-way ANOVA with Tukey’s post-test versus the sham group. **Figure S12.** Western blot analysis of IPTG-induced OMV^S1^ and SyBV^S1^ with anti-His Tag antibody. The filled triangle indicates the Lpp-OmpA-S1 complex. **Table S1.** Immunogenic outer membrane protein markers expressed in SyBV in comparison to OMV.

## Data Availability

The data that support the findings of this study are available from the corresponding authors upon reasonable request.

## Abbreviations

OMV: Outer membrane vesicles
SyBV: Synthetic bacterial vesicles
LPS: Lipopolysaccharide
TLR: Toll-like receptor
TEM: Transmission electron microscopy
TNF: Tumor necrosis factor
IL: Interleukin
PBS: Phosphate-buffered saline
BAL: Bronchoalveolar lavage
GO: Gene ontology
