# Supplementary figures and images for: Detoxified synthetic bacterial membrane vesicles as a vaccine platform against bacteria and SARS-CoV-2
Source: J Nanobiotechnology. 2023 May 19;21:156. doi: 10.1186/s12951-023-01928-w (PMC10196325; doi:10.1186/s12951-023-01928-w)

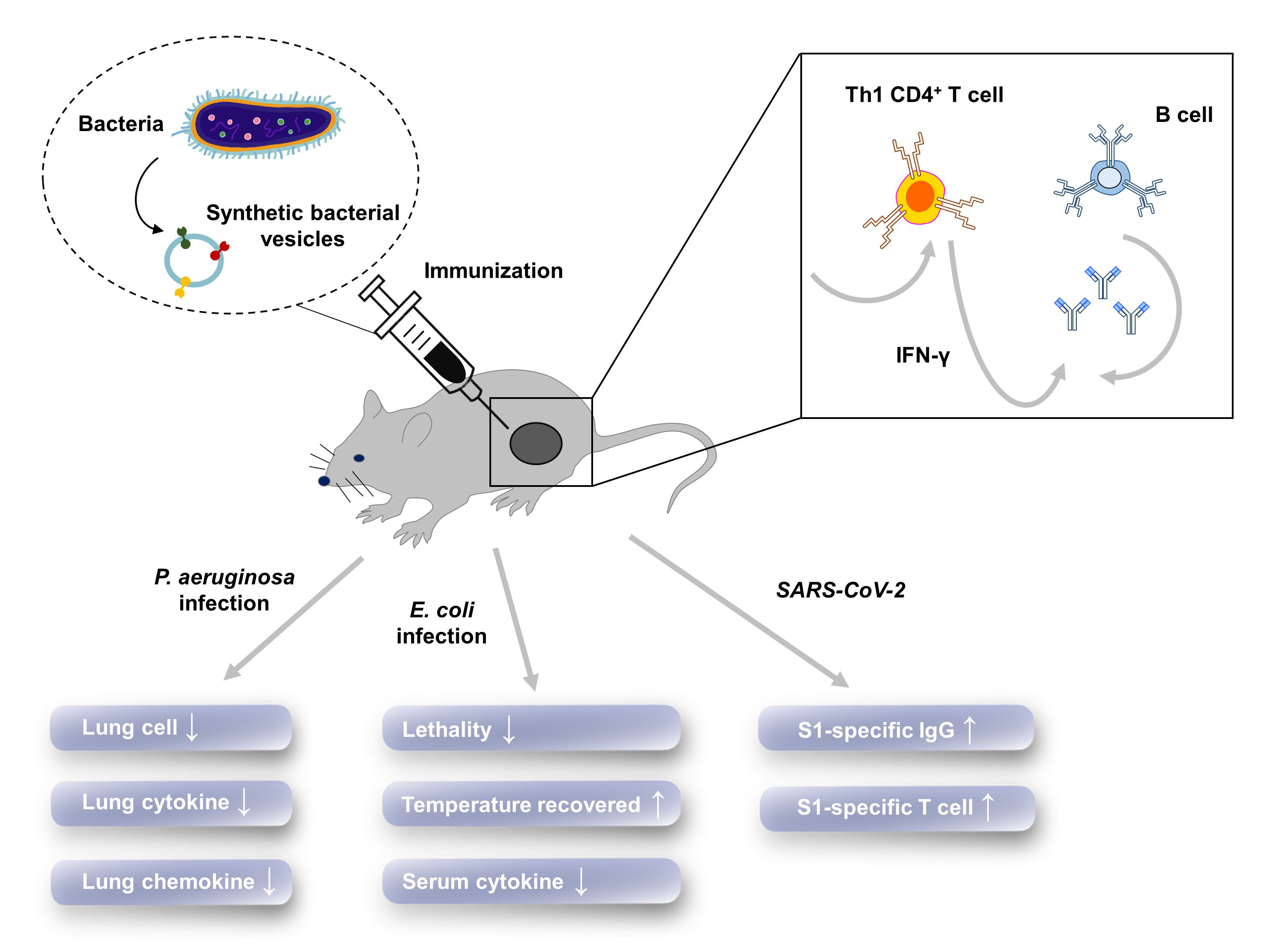

Supplement: Supplementary file 1 — Supplementary Material 1 [file 12951_2023_1928_MOESM1_ESM.jpg]
