# Supplementary material for: Detoxified synthetic bacterial membrane vesicles as a vaccine platform against bacteria and SARS-CoV-2
Source: J Nanobiotechnology. 2023 May 19;21:156. doi: 10.1186/s12951-023-01928-w (PMC10196325; doi:10.1186/s12951-023-01928-w)
Supplement: Supplementary file 2 — Additional file 1: figure S1: Schematic overview of OMV and SyBV isolation from cultured bacteria. Figure S2. Characterization of RNA isolated from OMV and SyBV. Representative electropherograms of RNA molecules isolated from SyBV in comparison to those from OMV. The filled triangle indicates internal marker. Figure S3. Quantification of DNA isolated from OMV and SyBV (n = 3 independent samples). Data are presented as the mean ± s.e.m. ns, not significant by unpaired two-tailed Student’s t-test. Figure S4. Comparison of protein composition between P. aeruginosa OMV and SyBV. (a) Principle component analysis of OMV and SyBV proteome. Two biological replicates per sample and three technical replicates for each biological replicate. (b) Partial view of heatmap of the hierarchical clustering based on the relative proteins abundance of OMV and SyBV proteome. Color code shows the normalized median abundance of proteins belonging to the category (red, most abundant; blue, least abundant). Figure S5. SyBV can be efficiently taken up by macrophages comparable to OMV. DiO-labelled vesicles (1 × 10^9^) were incubated with MH-S cells for 6 h. And, the uptake of SyBV by cells was compared with OMV by flow cytometry, and the results are shown as the percentage of DiO-positive cells (n = 3). Data are presented as the mean ± SEM. ***P < 0.001; ns, not significant, by one-way ANOVA with Tukey’s post test. Figure S6. Measurement of body temperature during immunization with P. aeruginosa OMV or SyBV. The temperature of mice was investigated at 24 h after each immunization (n = 5). Data are presented as mean ± s.e.m. ns, not significant; by one-way ANOVA with Tukey’s post test versus the sham group. Figure S7. Differential cell count and chemokine level in BAL fluid of mice immunized with P. aeruginosa OMV or SyBV. (a) The effect of OMV and SyBV on the numbers of BAL cells (macrophage, neutrophil and lymphocyte) was investigated at 48 h after the last challenge (n = 5). (b) The level of [file 12951_2023_1928_MOESM2_ESM.docx]

**
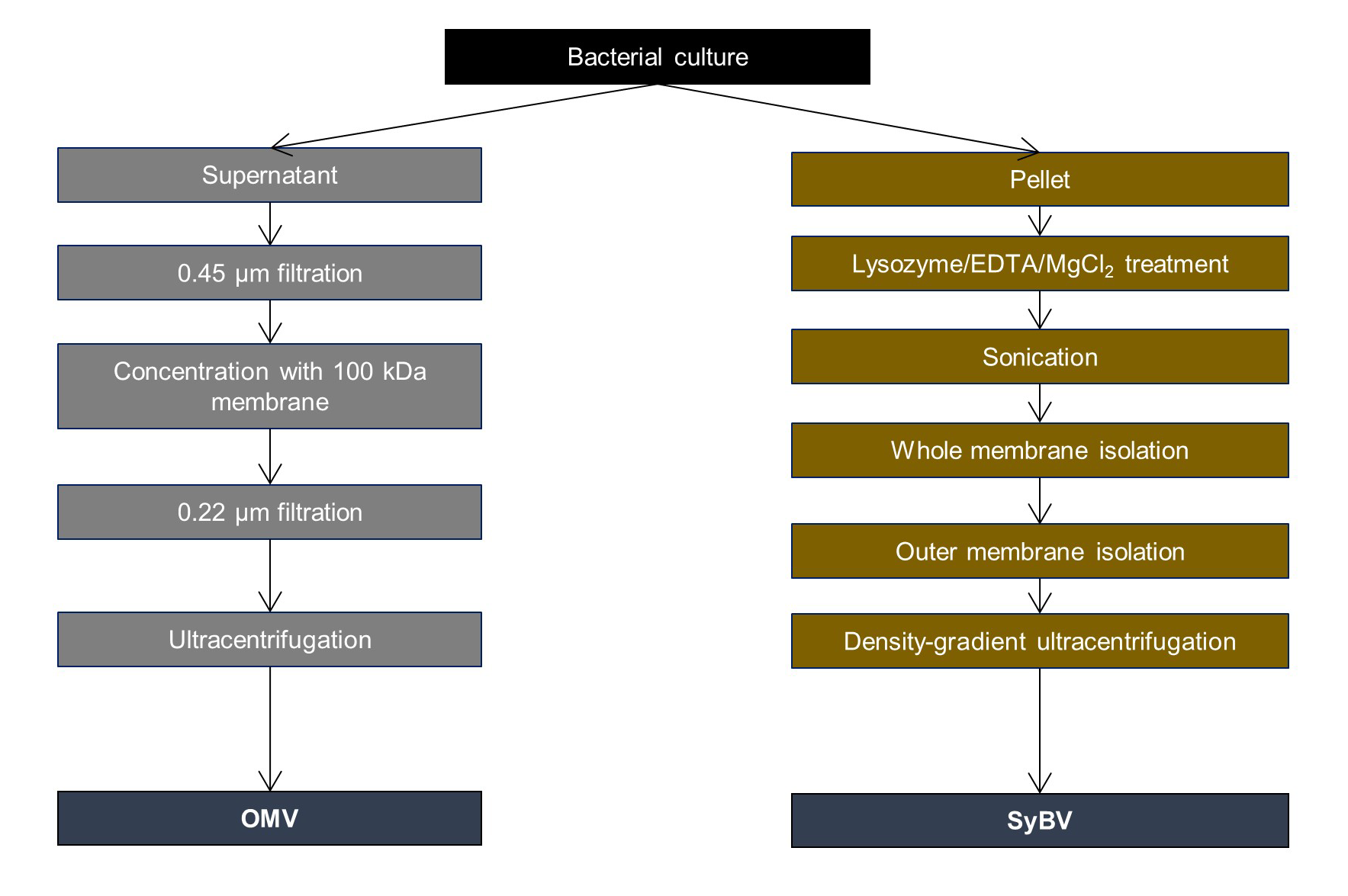
**

**Figure S1** Schematic overview of OMV and SyBV isolation from cultured bacteria.

**
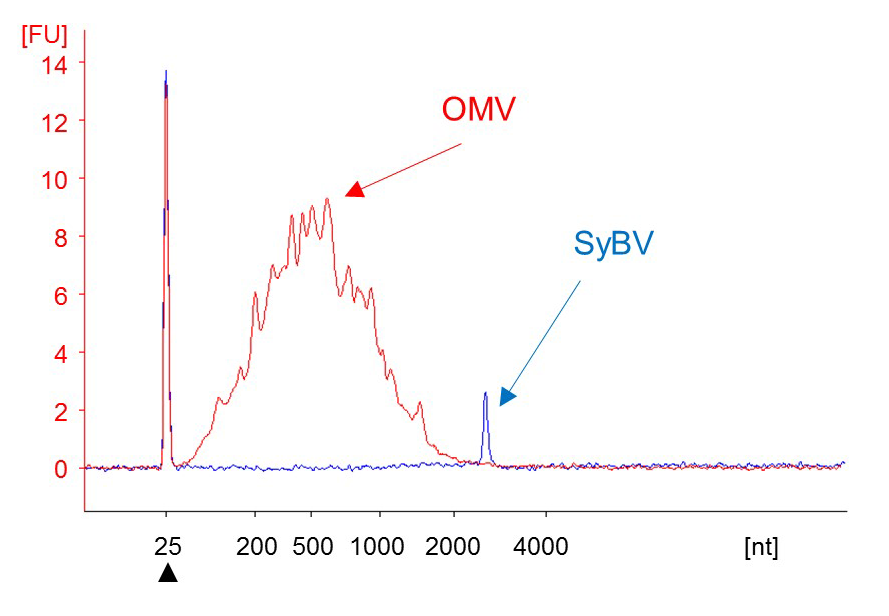
**

**Figure S2** Characterization of RNA isolated from OMV and SyBV. Representative electropherograms of RNA molecules isolated from SyBV in comparison to those from OMV. The filled triangle indicates internal marker.

**
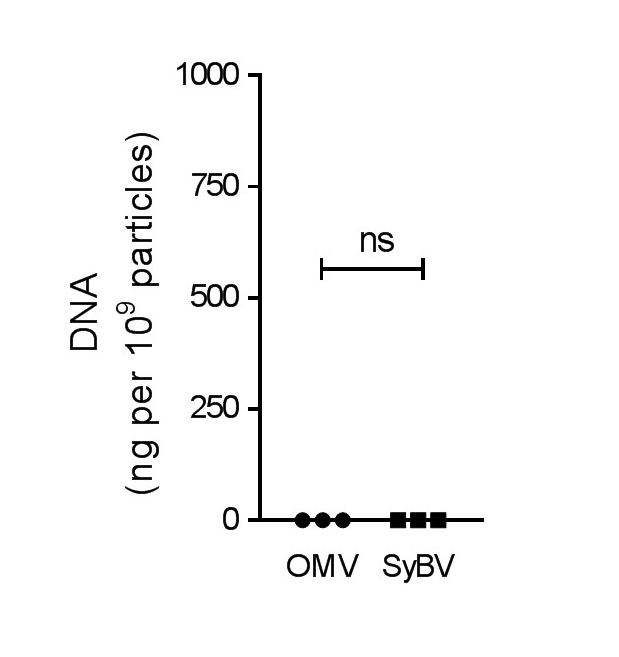
**

**Figure S3** Quantification of DNA isolated from OMV and SyBV (*n* = 3 independent samples). Data are presented as the mean ± s.e.m. ns, not significant by unpaired two-tailed Student’s *t*-test.

**
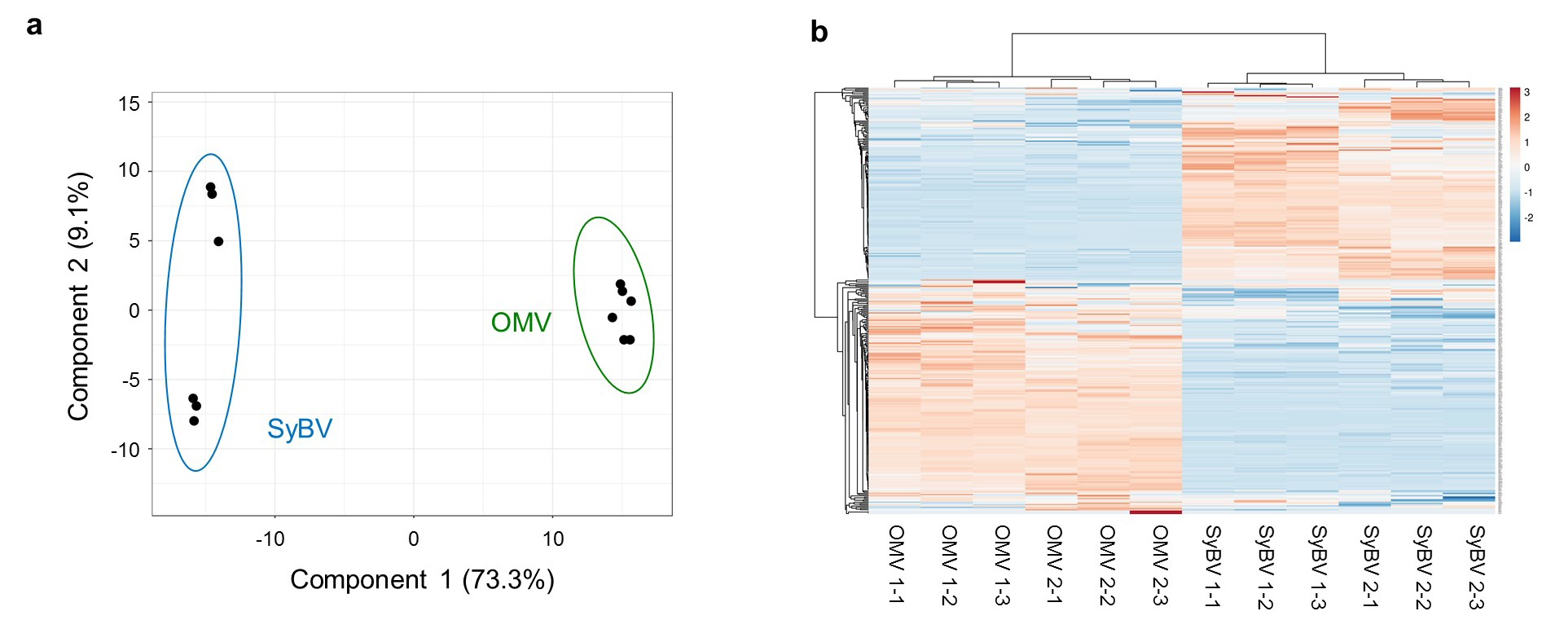
**

**Figure S4** Comparison of protein composition between *P. aeruginosa* OMV and SyBV. (**a**) Principle component analysis of OMV and SyBV proteome. Two biological replicates per sample and three technical replicates for each biological replicate. (**b**) Partial view of heatmap of the hierarchical clustering based on the relative proteins abundance of OMV and SyBV proteome. Color code shows the normalized median abundance of proteins belonging to the category (red, most abundant; blue, least abundant).


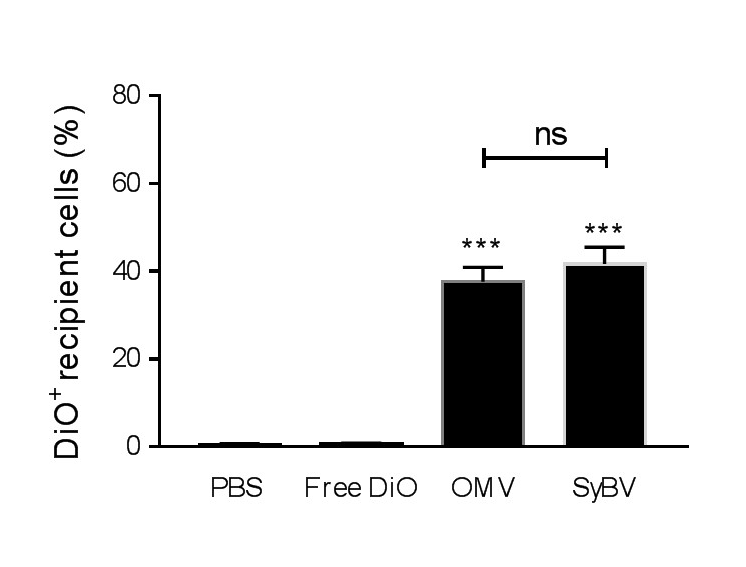


**Figure S5** SyBV can be efficiently taken up by macrophages comparable to OMV. DiO-labelled vesicles (1 × 10^9^) were incubated with MH-S cells for 6 h. And, the uptake of SyBV by cells was compared with OMV by flow cytometry, and the results are shown as the percentage of DiO-positive cells (*n* = 3). Data are presented as the mean ± SEM. ^***^*P* < 0.001; ns, not significant, by one-way ANOVA with Tukey’s post test.


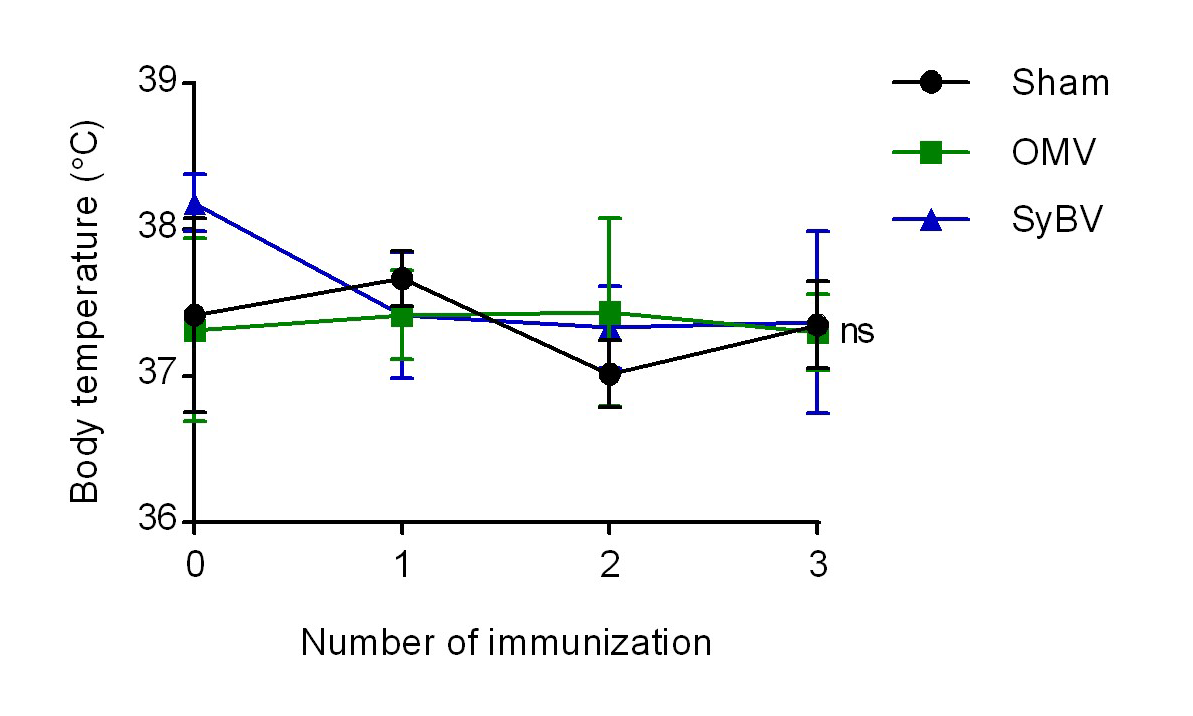


**Figure S6** Measurement of body temperature during immunization with *P. aeruginosa* OMV or SyBV. The temperature of mice was investigated at 24 h after each immunization (*n* = 5). Data are presented as mean ± s.e.m. ns, not significant; by one-way ANOVA with Tukey’s post test versus the sham group.

**
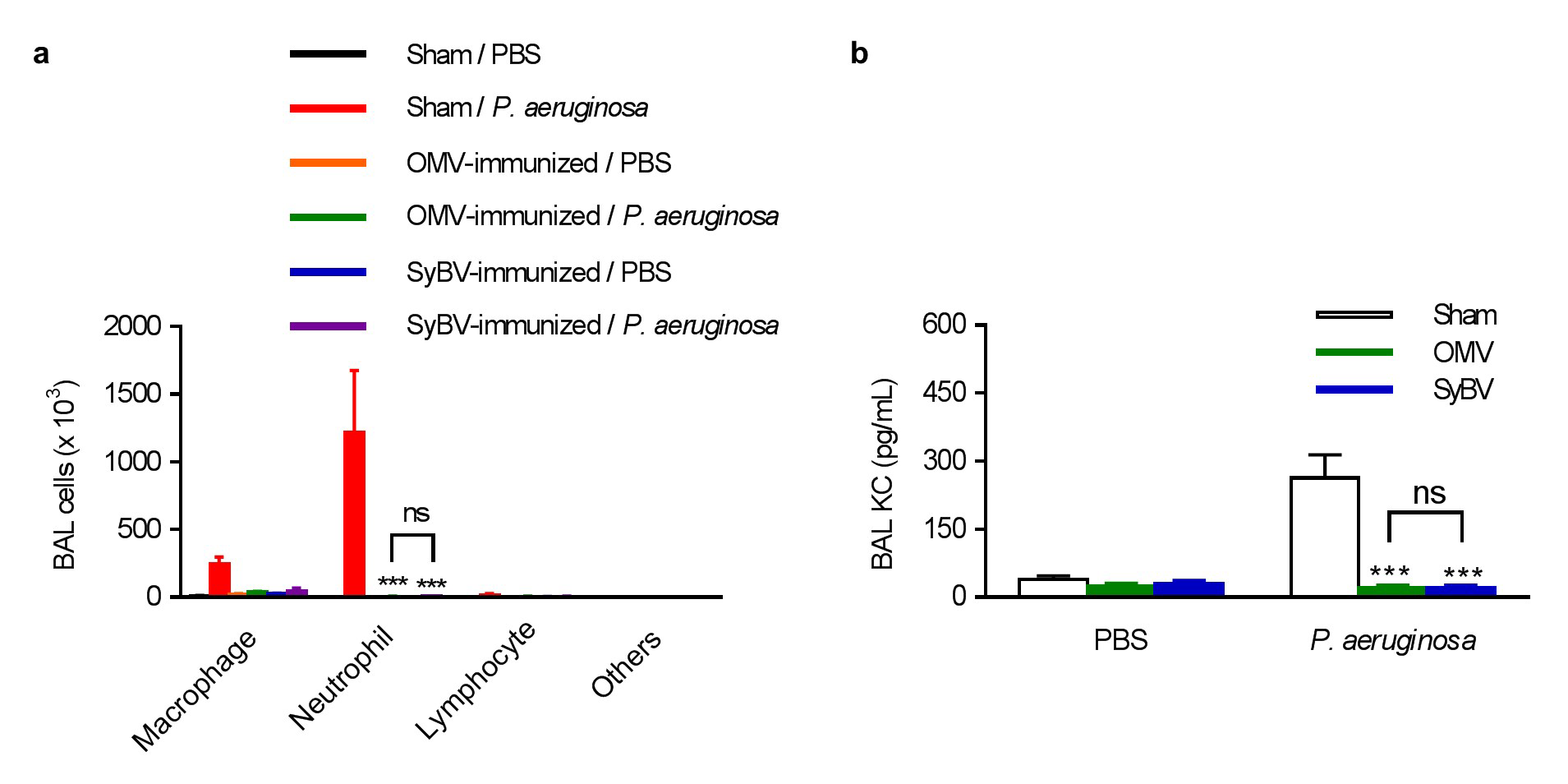
 Figure S7** Differential cell count and chemokine level in BAL fluid of mice immunized with *P. aeruginosa* OMV or SyBV. (**a**) The effect of OMV and SyBV on the numbers of BAL cells (macrophage, neutrophil and lymphocyte) was investigated at 48 h after the last challenge (*n* = 5). (**b**) The level of neutrophil-chemoattractant chemokine (KC) in BAL fluid was evaluated at 48 h after the last challenge (*n* = 5). Data are presented as mean ± s.e.m. ^***^*P* < 0.001; ns, not significant; by two-way ANOVA with Tukey’s post test versus the sham group.


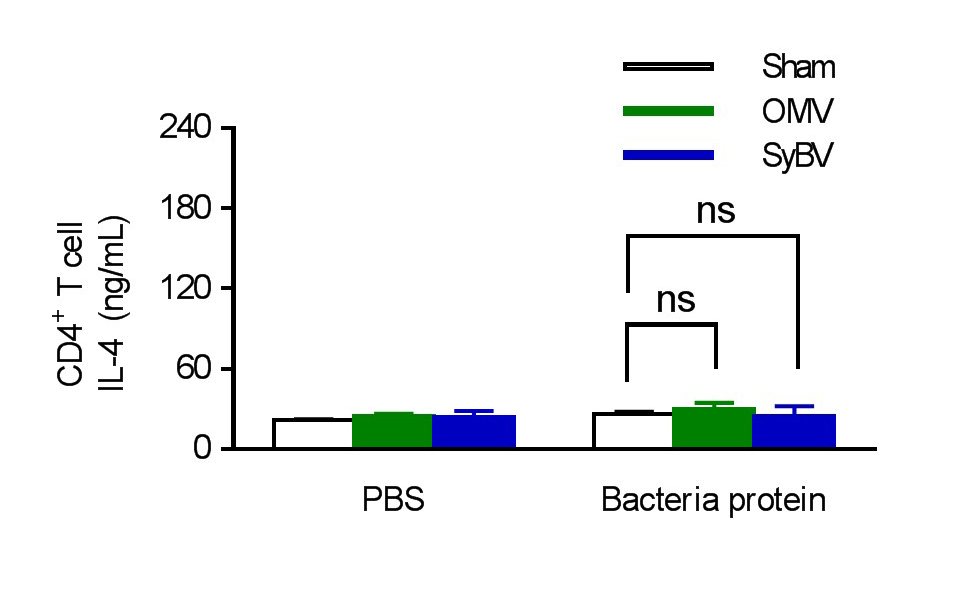


**Figure S8** The level of *P. aeruginosa*-specific CD4^+^ T-cell-derived IL-4 after CD4^+^ T-cells were isolated from immunized spleens (three independent samples). All data are presented as the mean ± s.e.m. ns, not significant by two-way ANOVA with Tukey’s post-test versus the sham group.

**
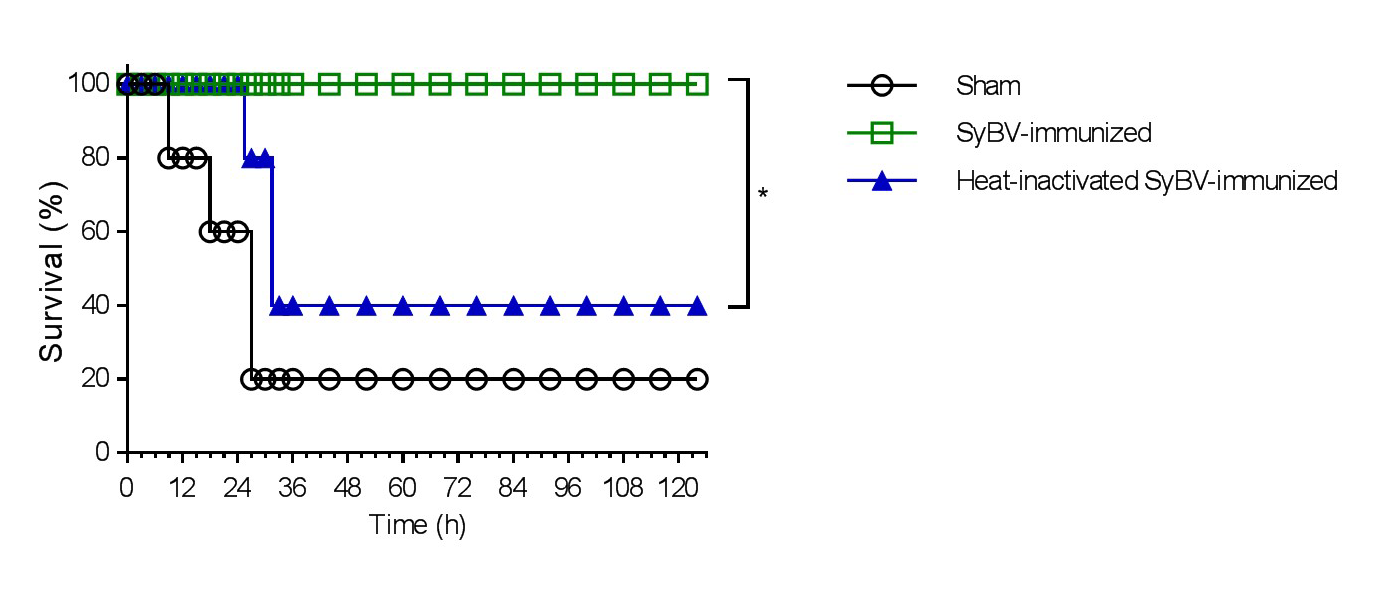
**

**Figure S9** Survival curve of mice immunized with heat-inactivated SyBV from *E. coli*. The result was monitored for 5 days after intraperitoneal challenge with lethal dose of *E. coli* (*n* = 10). For survival curve, log-rank (Mantel-Cox) test was used to compare with sham group (**P* < 0.05).

**
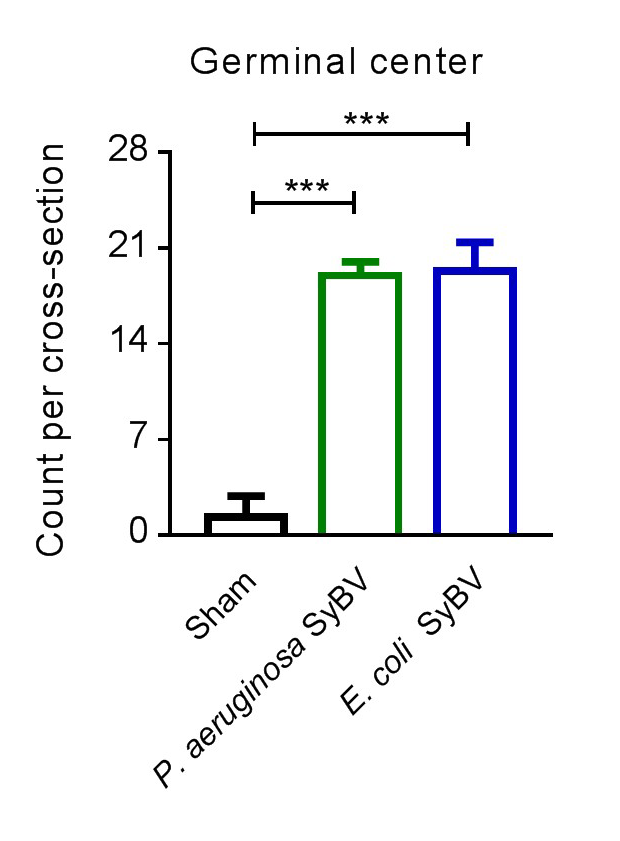
**

**Figure S10** Quantification of germinal center B cells. Graph shows cell counts per germinal center cross-section in the mice spleens immunized with *P. aeruginosa* or *E. coli* SyBV. Data are presented as mean ± s.e.m. ****P* < 0.001 by one-way ANOVA with Tukey’s post test.


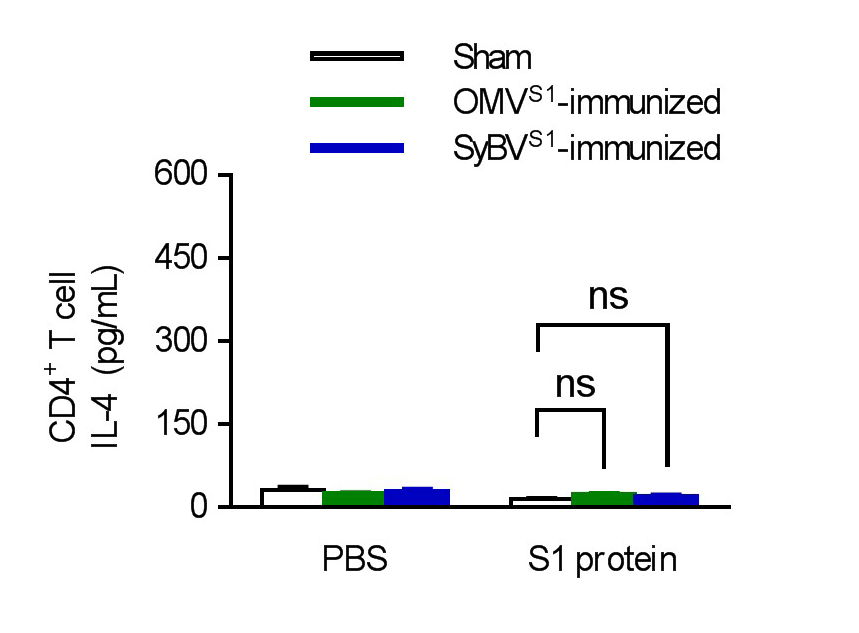


**Figure S11** The level of S1-specific CD4^+^ T-cell-derived IL-4 after CD4^+^ T-cells were isolated from immunized spleens (three independent samples). All data are presented as the mean ± s.e.m. ns, not significant by two-way ANOVA with Tukey’s post-test versus the sham group.

**
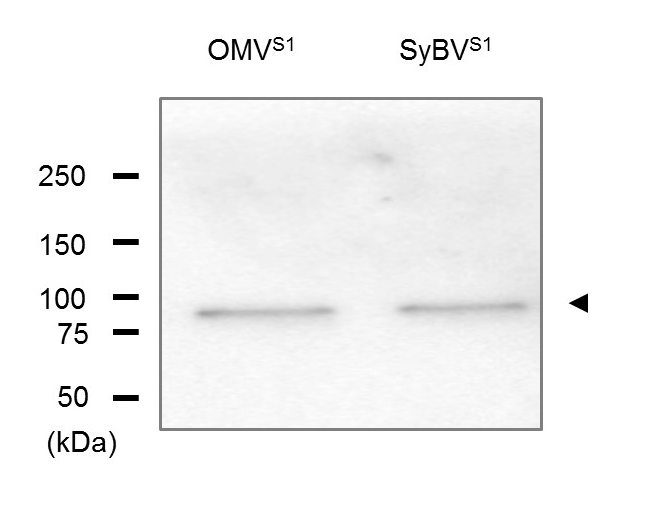
**

**Figure S12** Western blot analysis of IPTG-induced OMV^S1^ and SyBV^S1^ with anti-His Tag antibody. The filled triangle indicates the Lpp-OmpA-S1 complex.


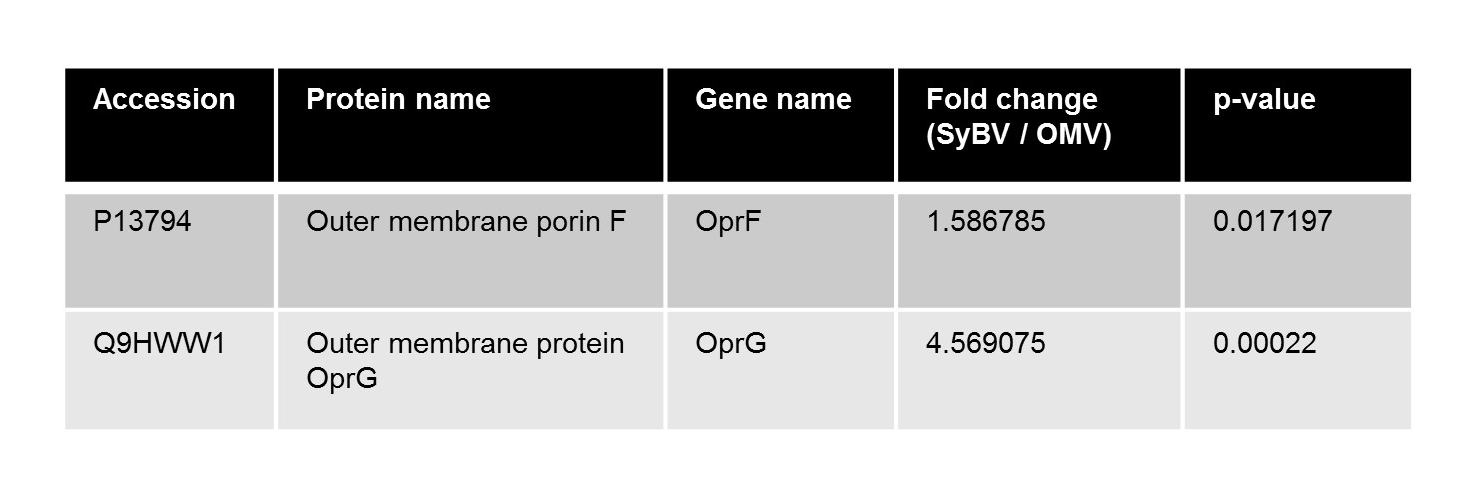


**Table S1** Immunogenic outer membrane protein markers expressed in SyBV in comparison to OMV.
